# Supplementary material for: IL-15 boosts mesothelin- and CD70-CAR NK cell potency in PDAC without added benefit from dual targeting
Source: Mol Ther Oncol. 2026 Aug 5;34(3):201316. doi: 10.1016/j.omton.2026.201316 (PMC13521009; doi:10.1016/j.omton.2026.201316)
Supplement: Supplementary file 1 [file mmc1.pdf]

OMTON, Volume *34*

Supplemental information

IL-15 boosts mesothelin- and CD70-CAR NK cell potency in PDAC without added benefit from dual targeting

Laura Gehrcken, Jasper Ott, Astrid Van den Eynde, Ho Wa Lau, Dana Liu, Christophe Hermans, Tias Verhezen, Stefanie Peeters, Carole Faghel, Philippe Joye, Gils Roex, Diana Campillo-Davo, Eva Lion, Filipe Elvas, Senada Koljenovic, Jorrit De Waele, Vera Hartman, Geert Roeyen, Hans Prenen, Filip Lardon, Christophe Deben, Evelien Smits, and Jonas Van Audenaerde

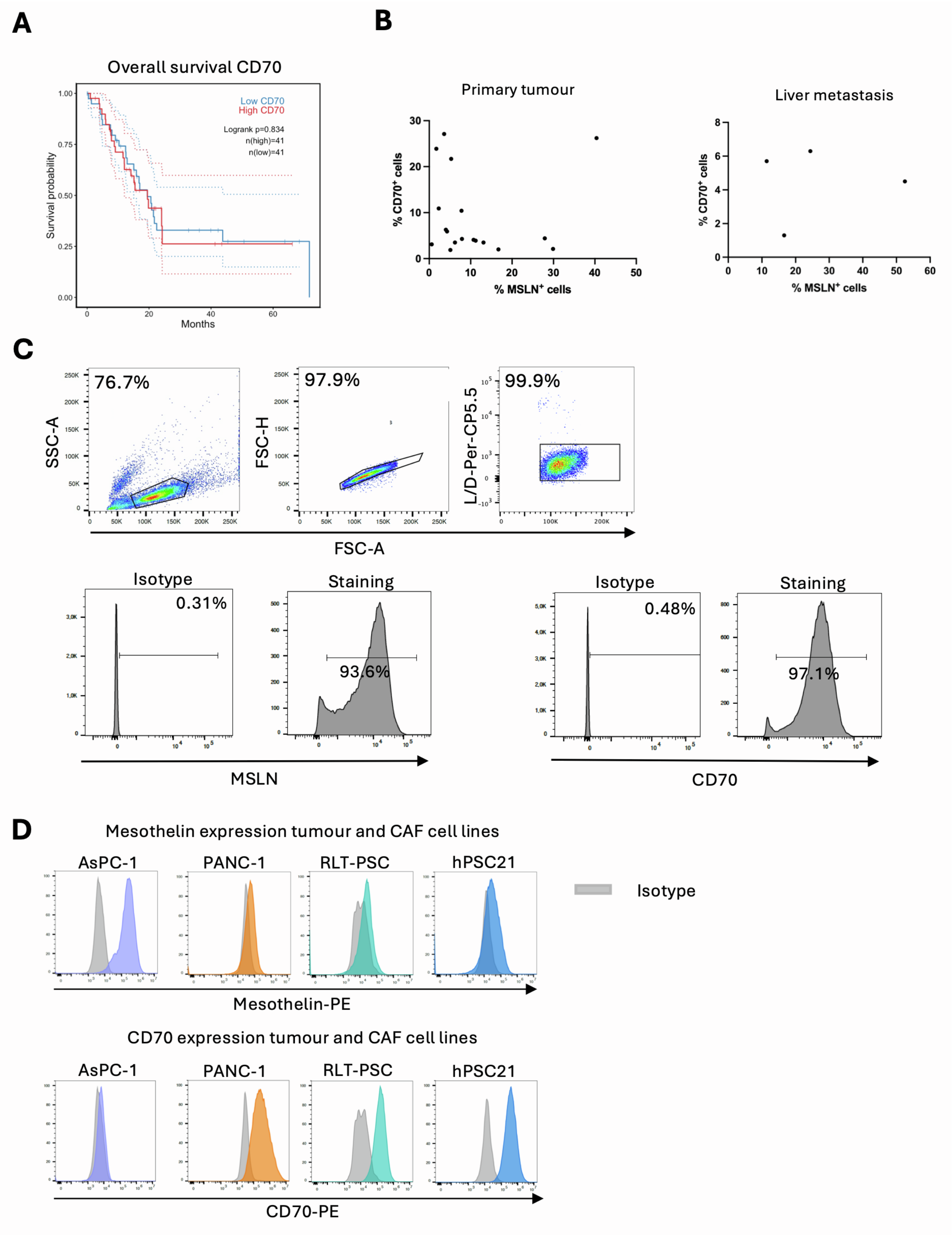

Figure S1 MSLN and CD70 expression in patient samples and PDAC cell lines. *(A) Kaplan-Meier analysis of TCGA-PDAC patients stratified by CD70 expression. Patients in the lower quartile were classified as CD70 low, while patients in the upper quartile were classified as CD70 high. Dotted lines represent 95% confidence intervals. Survival differences were assessed by using the log-rank test. (B) Scatter plot showing the percentage of CD70^{+} cells plotted against the percentage of MSLN^{+} cells across individual tumour (left) and liver metastasis (right) samples, not matched. (C) Representative flow cytometry plots depicting the gating strategy to determine CAR expression 24 h post-electroporation. NK-92 cells were gated for Singlets and then CAR expression analysis on live cells. (Bottom) Representative histograms for MSLN-CAR (left) and CD70-CAR (right) expression in comparison to the corresponding isotype. (D) Representative histograms of MSLN (top) and CD70 (bottom) on the four different cell lines AsPC-1, PANC-1, RLT-PSC and hPSC21 in comparison to the isotype control of the corresponding antibody.*

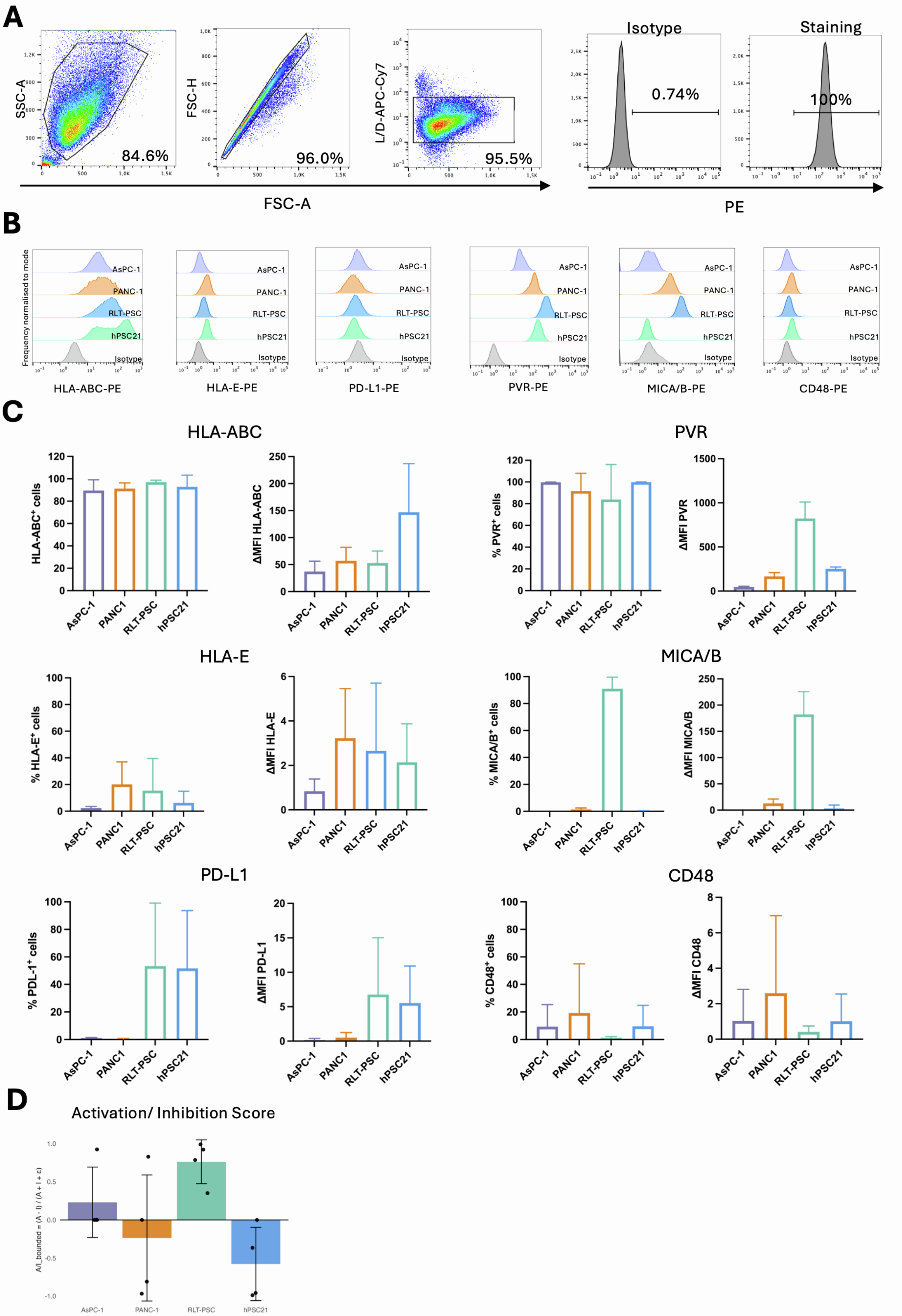

Figure S2 Expression of activating and inhibitory NK cell markers on PDAC tumour and CAF cell lines*. (A) Representative flow cytometry plots depicting the gating strategy to stain different tumour and CAF cell lines for inhibitory and activation marker expression. Tumour and CAF cells were gated for Singlets and then expression of the*

different markers was performed on live cells (left). Representative histograms showing the gating strategy to determine the positivity based on the isotype (right). (B) Representative overlay histograms showing the expression of the different markers (HLA-ABC, HLA-E, PD-L1, PVR, MICA/B, CD48) on the four different cell lines in comparison to the isotype (grey). (C) Individual bar plots for all markers (HLA-ABC; HLA-E; PD-L1, PVR, MICA/B, CD48 investigated on both % positive cells and mean fluorescence intensity minus FMO (fluorescence minus one) control. (D) Bounded antigen index across four cell lines (AsPC-1, PANC-1, RLT-PSC and hPSC21). Bars represent mean AI_bounded values with error bars indicating biological variability. AI_bounded is calculated as (A – I) / (A + I + ε), where A denotes NK-cell activation markers, I denotes inhibitory markers, and ε is a small constant. Positive AI_bounded values indicate a predominance of activation markers, values around 0 reflect a balance between activation and inhibition, and negative values indicate a higher proportion of inhibitory markers.

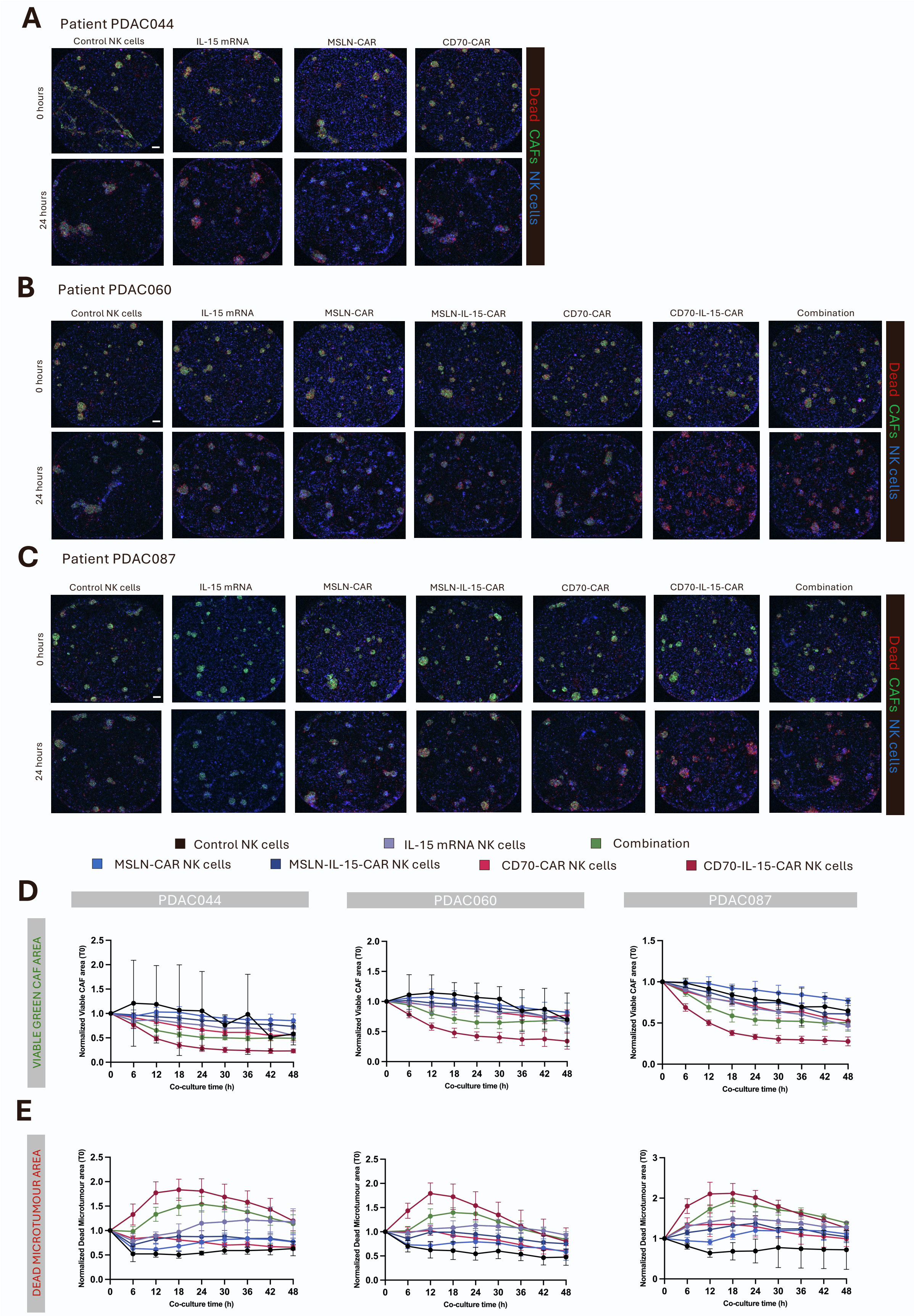

Figure S3 Representative images of PDAC microtumours and quantification of viable green area and dead microtumour area. *(A) Representative images of PDAC microtumours of PDAC044 at timepoint 0 and 24 h later after treatment with control, IL-15 mRNA, MSLN-CAR, CD70-CAR NK cells. Scale bar = 200 µm (B) Representative images of*

PDAC microtumours of PDAC060 at timepoint 0 and 24 h later after treatment with control, IL-15 mRNA, MSLN-CAR, MSLN-IL-15-CAR, CD70-CAR, CD70-IL-15-CAR NK cells or the combination. Scale bar = 200 µm (C) Representative images of PDAC microtumours of PDAC087 at timepoint 0 and 24 h later after treatment with control, IL-15 mRNA, MSLN-CAR, MSLN-IL-15-CAR, CD70-CAR, CD70-IL-15-CAR NK cells or the combination. Scale bar = 200 µm (D) Quantification of viable green CAF area over time in three different PDAC microtumours (PDAC044, PDAC060 and PDAC087). Curves depict area following treatment, normalized to timepoint 0 of each well. n = 3 (E) Quantification of dead microtumour area over time in three different PDAC microtumours (PDAC044, PDAC060 and PDAC087). Curves depict area following treatment, normalized to T0 of each well for each patient. n = 3

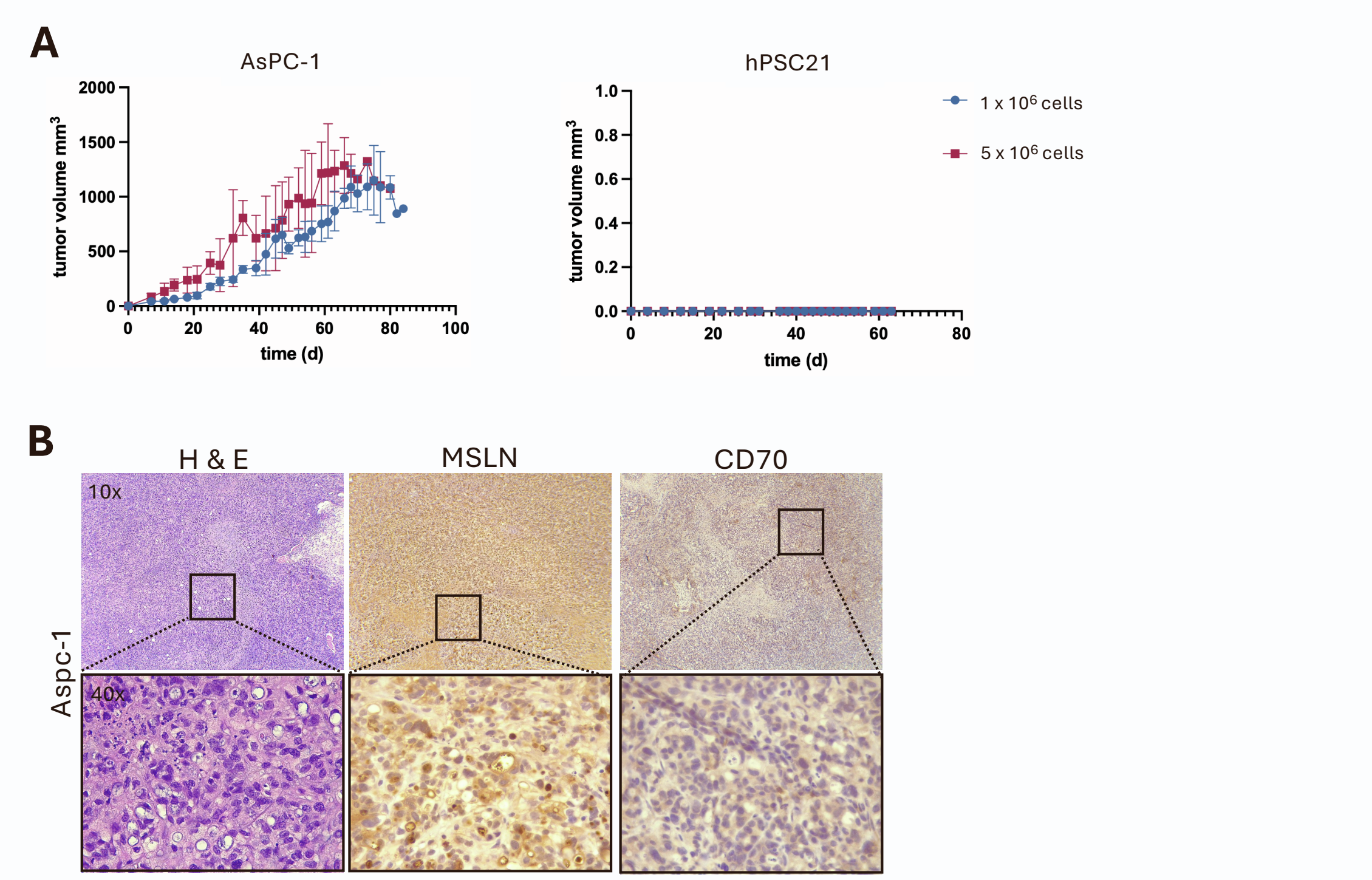

Figure S4 Pilot experiment to establish the mouse model *(A) Tumour growth of NSG mice inoculated with either 1 or 5 x 10*^{6} *AsPC-1 (left) or hPSC21 (right) respectively. (n = 3) (B) Representative IHC images of H&E, MSLN and CD70 staining of 1 x 10*^{6} *AsPC-1 bearing mice. (n = 3)*
