# Supplementary material for: IL-15 boosts mesothelin- and CD70-CAR NK cell potency in PDAC without added benefit from dual targeting
Source: Mol Ther Oncol. 2026 Aug 5;34(3):201316. doi: 10.1016/j.omton.2026.201316 (PMC13521009; doi:10.1016/j.omton.2026.201316)
Supplement: Supplementary file 2 [file mmc2.pdf]

Molecular Therapy
Oncology
Original Article

IL-15 boosts mesothelin- and CD70-CAR NK cell potency in PDAC without added benefit from dual targeting

Laura Gehrcken,[1] Jasper Ott,[1] Astrid Van den Eynde,[1] Ho Wa Lau,[1] Dana Liu,[1] Christophe Hermans,[1] Tias Verhezen,[1] Stefanie Peeters,[2,3] Carole Faghel,[2,3] Philippe Joye,[4] Gils Roex,[2] Diana Campillo-Davo,[2] Eva Lion,[2] Filipe Elvas,[4] Senada Koljenovic,[1,5] Jorrit De Waele,[1] Vera Hartman,[6] Geert Roeyen,[6] Hans Prenen,[7] Filip Lardon,[1] Christophe Deben,[1] Evelien Smits,[1,8] and Jonas Van Audenaerde[1,8]

[1]Center for Oncological Research (CORE), Faculty of Medicine and Health Sciences, University of Antwerp, 2610 Wilrijk, Belgium; [2]Laboratory of Experimental Haematology (LEH), Vaccine and Infectious Disease Institute (VAXINFECTIO), Faculty of Medicine and Health Sciences, University of Antwerp, 2610 Wilrijk, Belgium; [3]Department of Haematology, Antwerp University Hospital, 2650 Edegem, Belgium; [4]Molecular Imaging Center Antwerp (MICA), University of Antwerp, 2610 Wilrijk, Belgium; [5]Department of Pathology, Antwerp University Hospital, 2650 Edegem, Belgium; [6]Department of Hepatobiliary Transplantation and Endocrine Surgery, Antwerp University Hospital, 2650 Edegem, Belgium; [7]Department of Oncology, Multidisciplinary Oncology Center Antwerp, Antwerp University Hospital, 2650 Edegem, Belgium

Chimeric antigen receptor (CAR) therapies have shown great success in hematological malignancies but remain largely ineffective against solid tumors such as pancreatic ductal adenocarcinoma (PDAC). A key obstacle among various aspects, is the dense stromal barrier formed by cancer-associated fibroblasts (CAFs), providing a rationale for simultaneously targeting stroma and tumor cells. Using immunohistochemistry of primary PDAC tumors and liver metastases, we confirmed high mesothelin (MSLN) expression on tumor cells, and CD70 expression on tumor cells and predominantly CAFs. Based on these results and the favorable safety profile of CAR natural killer (NK) cells over CAR T cells, we generated MSLN- and CD70-targeting IL-15-armored CAR NK cells. Both constructs mediated cytotoxicity against different pancreatic cancer and CAF cell lines with varying antigen expression *in vitro*, demonstrating that both, the CAR-molecule and IL-15 were required to increase functionality against more treatment-resistant cell lines. Interestingly, pooled MSLN- and CD70-CAR NK cells did not significantly improve cytolysis compared to monotherapies in an advanced 3D *in vitro* model or *in vivo*. Together these findings highlight the limitations of dual-targeting approaches and underscore the need for advanced engineering strategies to improve CAR NK cells beyond antigen targeting and cytokine support in the PDAC microenvironment.

INTRODUCTION

Pancreatic ductal adenocarcinoma (PDAC) is one of the most aggressive malignancies with a 5-year survival rate of around 10%.[1] Its poor prognosis is primarily due to late-stage diagnosis and the limited amount of effective treatment options. Currently, systemic chemotherapy continues to be the standard-of-care for most patients, yet despite extensive research, therapeutic advances including immune checkpoint inhibitors (ICIs) and chimeric antigen receptor (CAR) T cells have shown limited clinical efficacy.[2,3] One of the reasons for this limited success is the immunosuppressive and desmoplastic tumor microenvironment (TME), characterized by a dense stromal barrier in which cancer-associated fibroblasts (CAFs) are a major cellular component. Simultaneously targeting this dense stromal barrier and tumor cells is a promising strategy for cellular therapies such as CAR natural killer (NK) cells.[4]

CAR NK cells offer several distinct advantages over CAR T cells for use in adoptive immunotherapy. While CAR T cells, especially directed against CD19 and BCMA, have transformed the treatment of hematologic malignancies, they entail severe toxicities, such as neurotoxicity or cytokine release syndrome (CRS) as well as high manufacturing costs.[5,6] In contrast, CAR NK cells mostly secrete IFN-γ and GM-CSF and less of the pro-inflammatory cytokines IL-6 or IL-10 that drive CRS and neurotoxicity, resulting in a safer clinical profile.[7–9] Another advantage of NK cells is their potential use in an allogeneic setting, allowing scalable production without the risk of graft versus host disease. Moreover, NK cells engage in multiple killing pathways such as natural or antibody-dependent cellular cytotoxicity, enabling response to target antigen loss or heterogeneity.[7] NK cells recognize tumor cells independently of antigen presentation via major histocompatibility complexes (MHCs).

Received 8 May 2026; accepted 3 August 2026;
https://doi.org/10.1016/j.omton.2026.201316.

[8]Senior author

Correspondence: J. Van Audenaerde, Center for Oncological Research (CORE), Faculty of Medicine and Health Sciences, University of Antwerp, 2610 Wilrijk, Belgium.
E-mail: jonas.vanaudenaerde@uantwerpen.be

Published by Elsevier Inc. on behalf of The American Society of Gene and Cell Therapy.
This is an open access article under the CC BY license (http://creativecommons.org/licenses/by/4.0/).

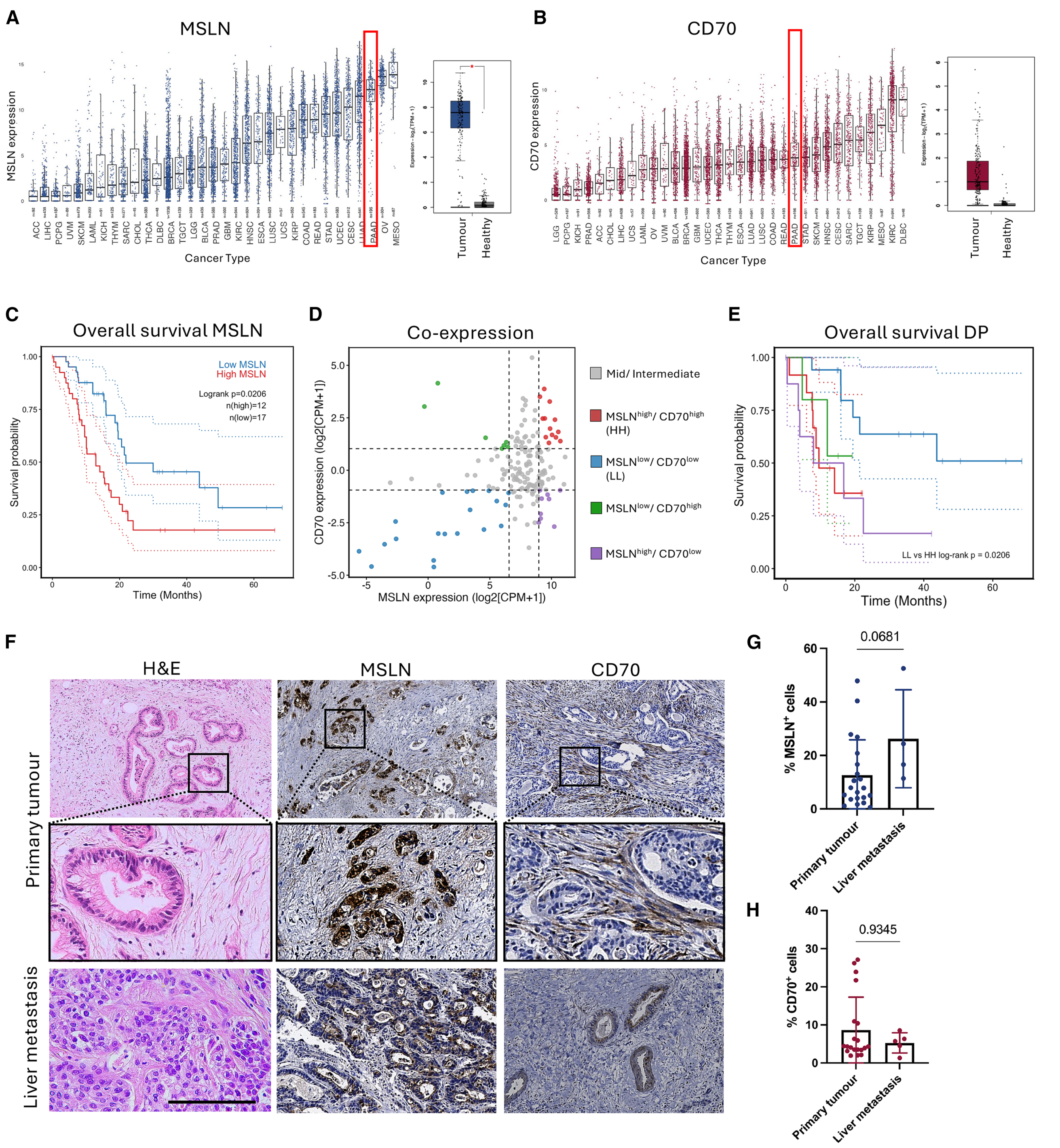

Figure 1. MSLN and CD70 as promising targets in PDAC

Pan-cancer expression profiling analysis of mesothelin (MSLN) (A) and CD70 (B) from 33 different cancer types obtained from publicly available bulk RNA-seq. from cBio Portal. TCGA-PAAD expression profiling of MSLN and CD70 between tumor samples and normal tissue displayed as log2(TPM+1). (C) Kaplan-Meier analysis of overall survival of TCGA-PAAD patients stratified by MSLN expression. Patients in the lower quartile were classified as MSLN low (n = 17) and patients in the upper quartile were classified as MSLN high (n = 12); the middle 50% were excluded. p values were determined by log rank test. Dotted lines indicated 95% confidence intervals. (D) Co-expression of MSLN and CD70 transcripts in TCGA-PAAD patients. Expression values are shown as log2(CPM+1). Patients were classified as low-low (bottom quartile for both), intermediate, $MSLN^{low}/CD70^{high}$, $MSLN^{high}/CD70^{low}$, or high-high (upper quartile for both). Dotted lines indicate the first and third quartile cutoffs. (E) Kaplan-Meier

(legend continued on next page)

Instead, they are regulated by a balance of activating and inhibitory receptors. As such, the "missing-self" mechanism allows them to target tumor cells that evade T cell recognition. This is particularly relevant in PDAC, where MHC-I downregulation is a common immune evasion feature and may increase tumor cell susceptibility to NK cell killing.[10] Building on these strengths, we designed two CAR NK cell constructs for efficient tumor and CAF killing.

Mesothelin (MSLN) is a glycosylated phosphatidylinositol-anchored protein highly overexpressed in several malignant tissues such as mesothelioma, ovarian, or pancreatic cancer, while it is only expressed in low amounts on healthy cells, such as mesothelial cells lining the pleura or pericardium. Additionally, MSLN has been associated with chemotherapy resistance and promotion of PDAC tumor development.[11] This makes MSLN a prime target for cellular therapies with limited off-tumor toxicity.[12–16] Additionally, our lab previously identified CD70 as a pan-cancer target and demonstrated that its expression is associated with tumor promoting characteristics in colorectal cancer, suggesting a broader role for other solid tumors as well.[17,18] CD70 is a tumor necrosis factor superfamily ligand for CD27, regulating the survival and co-stimulation of T, B, and NK cells, transiently expressed on activated immune cells.[17,19] In PDAC, CD70 is expressed on a subset of tumor cells and CAFs within the same lesion.[18] Elevated CD70 levels were associated with worse survival among adjuvant chemotherapy receiving patients and contributed to an immunosuppressive TME.[20] Consequently, multiple MSLN- and CD70-directed therapies such as CAR T cells or monoclonal antibodies (e.g., anetumab-ravtansine[21] or cusatuzumab,[22] respectively) have demonstrated acceptable safety profiles.[16,17] Additionally, for MSLN, T cell receptor fusion construct consisting of a single domain anti-MSLN antibody integrated into a T cell receptor. A clinical phase I/II trial was safe in solid tumor patients.[12] Together, the tumor-restricted expression and functional relevance of MSLN and CD70 provide rationale for dual targeting as a next-generation immunotherapy designed to simultaneously reduce the stromal barrier by targeting $CD70^+$ CAFs and eradicate $MSLN^+$ tumor cells.

In this study, using RNA-sequencing data from the The Cancer Genome Atlas (TCGA) database as well as immunohistochemical analysis of primary PDAC tumors and liver metastases, we highlighted both MSLN and CD70 as promising therapeutic candidates for dual targeting. Moreover, we evaluated the efficacy of MSLN- and CD70-targeting CAR NK cells and the importance of IL-15 for those NK cells on different PDAC cancer and CAF cell lines as well as patient-derived microtumors. In addition, we explored the potential benefit of pooled administration of both MSLN- and CD70-targeting CAR NK cells. As we only observed a rather modest additive effect, our data opens the debate on the necessity of dual-targeting strategies using CAR NK cells versus increased risks of on-target off-tumor toxicity.

RESULTS

CD70 and MSLN as promising targets in PDAC

We first evaluated the clinical relevance of MSLN and CD70 in PDAC. To compare MSLN and CD70 expression across different cancer types, we conducted a pan-cancer analysis using publicly available data. The bulk RNA-seq data from the TCGA PanCancer Atlas demonstrated varying levels of both MSLN and CD70 expression across 33 cancer types (Figure 1A). PDAC is among the tumor types with the highest MSLN-expression, while CD70 expression is intermediate (Figure 1B). In a follow-up analysis of the publicly available TCGA dataset, we compared both MSLN and CD70 RNA expression within PDAC to healthy pancreatic tissue samples using Gepia2.[23] Here, MSLN expression was significantly higher on the tumor compared to healthy tissue, whereas CD70 was elevated in tumors, however not significantly (Figures 1A and 1B). Higher MSLN expression in PDAC patients could also be linked to increased patient overall survival compared to those with low MSLN expression (log rank $p = 0.0206$) (Figures 1C and S1A), whereas no survival difference was seen for CD70. Notably, as previously reported by our group, TCGA-PAAD data revealed that CD70 mRNA expression correlated with CAF gene signatures, including a transcriptional program associated with immune-checkpoint blockade resistance.[18] To further investigate the relationship between MSLN and CD70, a co-expression analysis was performed (Figure 1D). MSLN and CD70 transcript levels demonstrated heterogeneity across the investigated cohort, allowing differentiation into five distinct groups: Low-Low (LL, <Q1 for both, blue, n = 17/178), High-High (HH, >Q3 for both, red, n = 12/178), $MSLN^{low}/CD70^{high}$ (green, n = 5/178), $MSLN^{high}/CD70^{low}$ (purple, n = 9/178), and the intermediate ones (gray). This analysis revealed a subset characterized by the concurrent high expression of MSLN and CD70. Consistent with this, survival analysis demonstrated that patients with high levels of both markers (HH), had a significantly worse overall survival compared to patients exhibiting low expression levels (LL) (log rank p =0.0206) (Figure 1E). In contrast, patients with discordant expression patterns ($MSLN^{low}/CD70^{high}$ and $MSLN^{high}/CD70^{low}$) did not differ significantly from the HH group. Notably, MSLN-high tumors tended to show poorer overall survival irrespective of CD70 expression status, with both MSLN-high/CD70-high and MSLN-high/CD70-low subgroups displaying less favorable survival compared to their MSLN-low counterparts. This suggests that MSLN expression may contribute to adverse outcome beyond CD70 status, although this observation should be treated with caution due to the low subgroup size and heterogeneity. To further validate the

analysis of overall survival comparing low MSLN/high CD70, high MSLN/low CD70, low MSLN/low CD70, and high MSLN/high CD70 TCGA-PAAD groups based on quartile-defined co-expression. p values were determined using log rank test. (F) Representative images of H&E, MSLN, and CD70 staining of a PDAC patient on primary tumor and liver metastasis (not matched). Top and bottom row scale bars, 100 μm. Middle row scale bars, 50 μm. Distribution of $MSLN^+$ (G) and $CD70^+$ cells (H) found in the TME of PDAC patients, either in the primary tumor (n = 23) or liver metastasis (n = 4–5). Statistical significances were assessed by using Mann-Whitney U test. *$p < 0.05$; DP, double-positive.

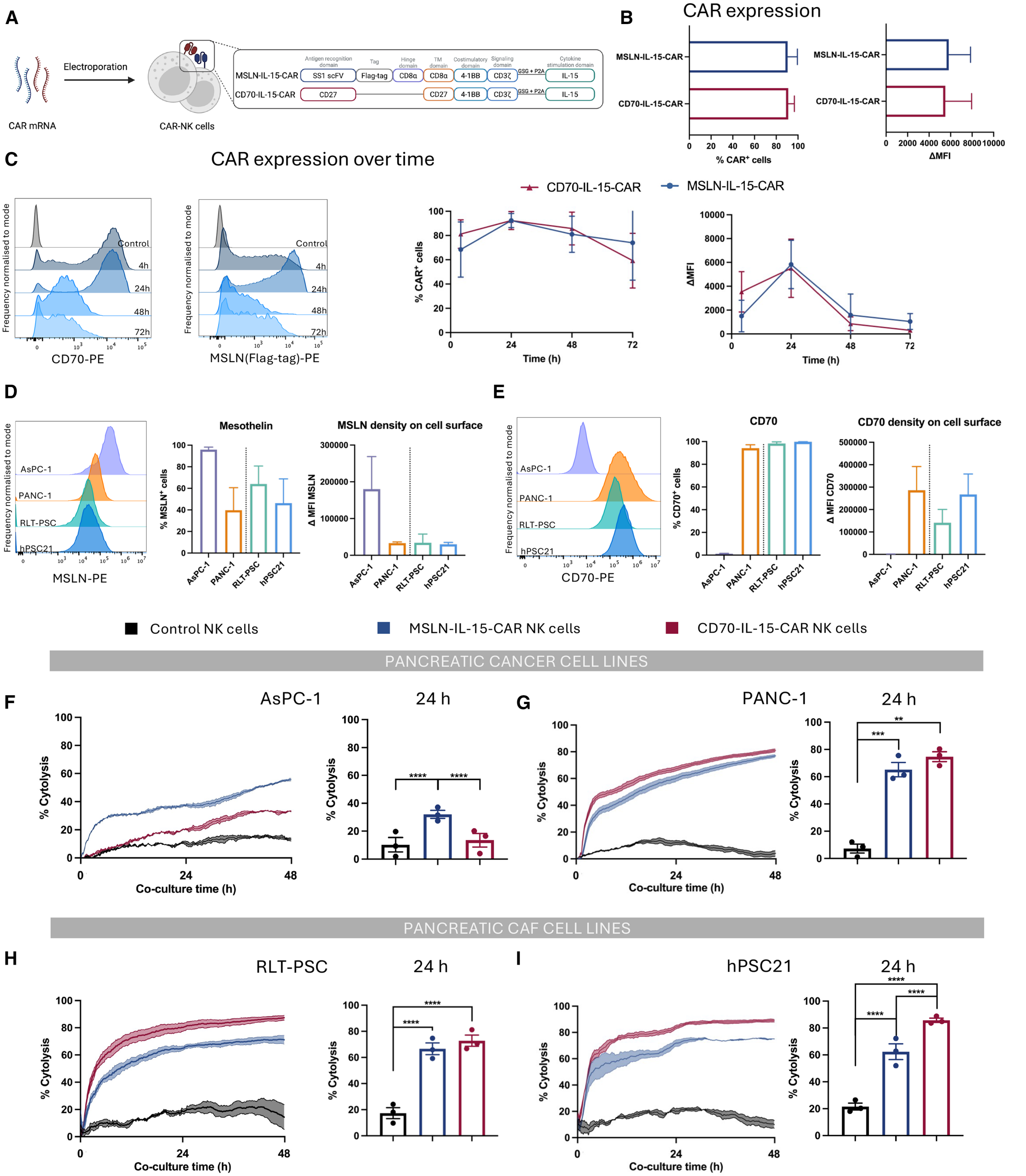

Figure 2. MSLN-IL-15 and CD70-IL-15-CAR NK cell constructs demonstrated targeted cytotoxicity against pancreatic cancer cell and CAF cell lines

(A) Structural composition of the mesothelin-IL-15 (MSLN) CAR and CD70-IL15-CAR. (B) MSLN-IL-15-CAR and CD70-IL-15-CAR expression was detected on the surface of NK-92 cells using flow cytometry 24 h after electroporation ($n = 6$). The graph on the left depicts the amount of CAR^{+} cells while the right graph shows the intensity of CAR expression depicted as mean fluorescence intensity minus isotype control (ΔMFI). (C) Representative histograms show MSLN-IL-15-CAR or CD70-IL-15-CAR expression 4,

(legend continued on next page)

expression of MSLN and CD70 on a protein level, we investigated a cohort of PDAC patient samples from 28 primary tumors and 5 liver metastases stained for H&E, MSLN, or CD70 expression using IHC. Both primary tumor and liver metastasis demonstrated MSLN and CD70 expression analyzed by intensity of DAB staining. In primary tumors, MSLN expression was more confined to the tumor cells, whereas CD70 expression was mainly observed on CAFs, but also on tumor cells (Figures 1F–1H). The primary tumor exhibited a mean % of MSLN positive cells of 12.6, compared to 8.6% for $CD70^+$ cells in the primary tumor. In liver metastasis, the mean proportion of $MSLN^+$ cells was 26.2% compared to 5.3% for CD70 (Figures 1H and 1I). These differences were not significant. Taken together, these results indicate that both targets merit further investigation in a dual CAR NK cell approach, as they are expressed on tumor cells and CAFs. Furthermore, we observed inter-patient heterogeneity in MSLN and CD70 expression, in line with TCGA data analysis (Figures 1D–1G, 1H, and S1B). Specifically, certain patients displayed high MLSN with low CD70 levels, while others demonstrated the inverse profile across both the primary tumor lesions and the liver metastasis (Figure S1B).

CD70- and MSLN-targeting CAR NK cells demonstrate PDAC killing *in vitro*

Based on the MSLN and CD70 expression data in PDAC, we generated two fourth-generation CAR constructs, noting that the CD70 CAR construct has already been described elsewhere.[18] Shortly, the single chain fragment variable (scFv) of the MSLN-CAR is based on SS1P, an anti-MLSN immunotoxin whereas the CD70-CAR is based on the natural receptor, CD27 (Figure 2A). Electroporation of NK-92 cells with MSLN-IL-15- and CD70-IL-15-CAR mRNA resulted in ∼90% CAR^+ cells for both CAR constructs (MSLN-IL-15-CAR: 87.9 ± 10.1%; CD70-IL-15-CAR: 89.5 ± 6.3%) (Figures 2B and S1C). CAR expression over time revealed the highest expression 24 h after electroporation (MSLN-IL-15-CAR: 90.5% ± 4%, $\Delta MFI_{MSLN\text{-}IL\text{-}15\text{-}CAR}$: 5,826.7 ± 1171.6; CD70-IL-15-CAR: 91.1 ± 2.5%, $\Delta MFI_{CD70\text{-}IL\text{-}15\text{-}CAR}$: 5,508.4 ± 1411.5) which decreased until 72 h (Figure 2C). To examine their suitability for CAR-specific cytotoxicity, different PDAC and CAF cell lines were analyzed for MSLN and CD70 expression using flow cytometry (Figures 2D, 2E, and S1D). Cancer cells demonstrated high MSLN expression on AsPC-1 (95.8 ± 4.2%, ΔMFI_{PE} 180,172.7 ± 153,358.5), low MSLN expression on PANC-1 (39.7 ± 36.3%, ΔMFI_{PE} 33,114.7 ± 5,632.3), while AsPC-1 were $CD70^{low}$ (1.2 ± 0.6%, ΔMFI_{PE} 1,445.3 ± 629.6) and PANC-1 $CD70^{high}$ (94.2 ± 5.3%, ΔMFI_{PE} 286,196.7 ± 183,205.1). CAF cell lines RLT-PSC and hPSC21 showed low to moderate MSLN expression (RLT-PSC: 64.1% ± 28.8%, ΔMFI_{PE} 34,004.7 ± 41,176.9; hPSC21: 46.3 ± 38.9%, ΔMFI_{PE} 29,904.7 ± 8,983.2). hPSC21s (99.8 ± 0.1%, ΔMFI_{PE} 267,432 ± 157,698) displayed high CD70 expression, while RLT-PSC demonstrated moderate CD70 expression (98.3 ± 2.6%, ΔMFI_{PE} 140,930 ± 103,507.1) (Figures 2D, 2E, and S1D). Using those cell lines enabled us to evaluate CAR NK cell cytotoxicity against both high and low antigen expressing tumor cells. All four cell lines were co-cultured with either control NK cells, MSLN-IL-15-CAR or CD70-IL-15-CAR NK cells and longitudinal % cytolysis was determined from real-time impedance measurements. In line with the MSLN expression data on AsPC-1, the anti-MSLN-IL-15-CAR NK cells displayed significantly higher cytolysis of AsPC-1 cells (32 ± 2.8%) compared to the CD70-IL-15-CAR (13.5 ± 4.9%) or the control condition (10.3 ± 5.2%) (Figure 2F). The anti-CD70-IL-15-CAR NK cells killed PANC-1, RLT-PSC, and hPSC21 (PANC-1: 74.7 ± 3.6%; RLT-PSC: 72.8 ± 4.3%; hPSC21: 85.7 ± 1.6%) significantly better than the control NK cells (PANC-1: 7.3 ± 3.2%; RLT-PSC: 17.3 ± 4.2%; hPSC21: 21.6 ± 2.5%). However, the MSLN-IL-15-CAR NK cells demonstrated significantly increased cytotoxicity against MSLN intermediate or $MSLN^{low}$ cell lines (PANC-1: 65.2 ± 5.2%; RLT-PSC: 66.6 ± 4.5%; hPSC21: 62.3 ± 5.8%) compared to the control. Nevertheless, CD70-IL-15-CAR NK cells killed hPSC21 in co-culture significantly better than the MSLN-IL-15-CAR NK cells (Figure 2G). Here, we demonstrated that both CAR constructs were surface-expressed on NK-92 for up to 72 h post-electroporation and significantly increased lysis of PDAC tumor cell and CAF cell lines versus the control.

IL-15 boosts CAR NK cell lysis and improves activity against resistant cell lines

We observed that MSLN-IL-15-CAR NK cells eradicated tumor and CAF cells at comparable levels as CD70-IL-15-CAR NK cells across $MSLN^{low}$ cell lines, which led us to investigate the role of IL-15 within the CAR construct. To assess the individual contribution of CAR signaling and to confirm that IL-15 enhances CAR-mediated NK cell cytotoxicity in PDAC, the IL-15 sequence was removed from the CAR constructs (Figure 3A). Analysis of CAR expression 24 h post-electroporation revealed that inclusion of IL-15 in the CAR construct enhanced MSLN-IL-15-CAR expression (80.4 ± 15.5%, ΔMFI: 889,245.7 ± 1,006,185.7), compared to the MSLN-CAR lacking IL-15 (41.1 ± 11.1%, ΔMFI: 56,430.7 ± 22,224.2) (Figure 3B). However, no significant difference was observed between the CD70-CAR with (95.2 ± 2.6%, ΔMFI: 501,512.8 ± 256,879.1) and

24, 48, and 72 h after electroporation (gray = isotype control). The graphs on the right represent the CAR expression over time by depicting % of CAR^+ cells or the ΔMFI ($n = 3$). (D) (Left) Representative flow cytometry histograms of MSLN expression on tumor (AsPC-1, PANC-1) or CAF (RLT-PSC, hPSC21) cell lines. (Right) Quantification of percentage of $MSLN^+$ cells and intensity of MSLN expression (ΔMFI) for AsPC-1, PANC-1, RLT-PSC, and hPSC21. (E) (Left) Representative flow cytometry histograms of CD70 expression on tumor (AsPC-1, PANC-1) or fibroblast (RLT-PSC, hPSC21) cell lines. (Right) Quantification of percentage of $CD70^+$ cells and intensity of CD70 expression (ΔMFI) for AsPC-1, PANC-1, RLT-PSC, and hPSC2. (F&G) Cytolysis of pancreatic cancer cell lines (AsPC-1 and PANC-1) (F) and CAF cell lines (RLT-PSC and hPSC21) (G). Representative graph of longitudinal cytolysis follow-up with the xCELLigence RTCA system using the Immunotherapy module and the corresponding quantification 24 h after adding the CAR NK cells to the co-culture with NK cells (control), MSLN-IL-15-CAR and CD70-IL-15-CAR NK cells. Error bars represent standard error of the mean (SEM) cytolysis (n =3). Linear mixed models with Tukey's correction for multiple comparisons were applied to compare the means of cytolysis. *p <0.05, **p <0.01, ***p <0.001, ****p <0.0001. Error bars represent the mean values ± standard deviation unless described otherwise. Each dot represents one biological repeat.

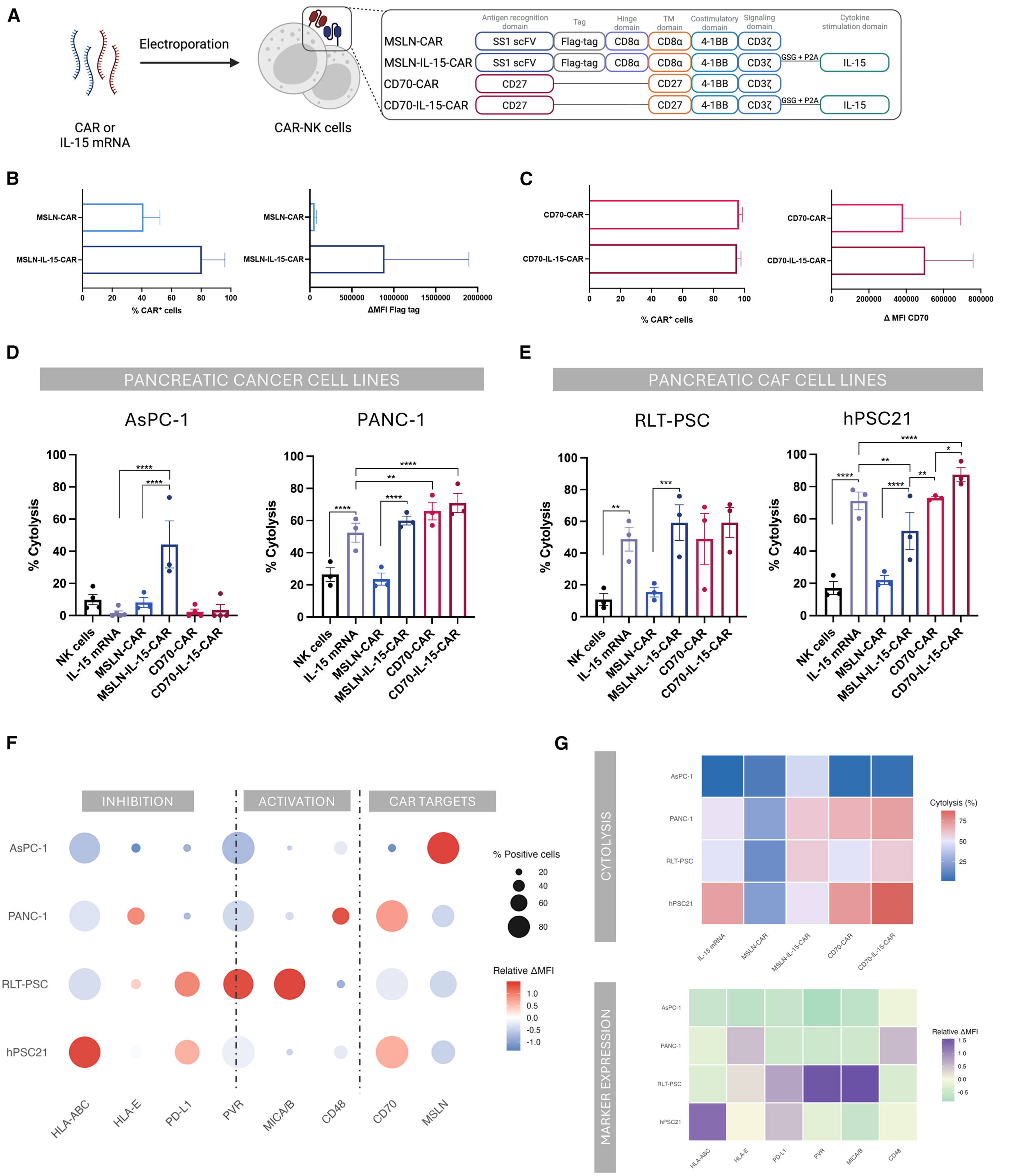

Figure 3. Addition of IL-15 to the CAR constructs enhanced cytotoxicity against more resistant cell lines

(A) Structural composition of the MSLN-IL-15-CAR, MSLN-CAR and CD70-IL15-CAR, CD70-CAR. (B) MSLN-IL15-CAR and MSLN-CAR expression on the surface of NK-92 cells was measured using flow cytometry 24 h after electroporation ($n = 3$). The graph on the left depicts the amount of CAR⁺ cells while the right graph shows the intensity of CAR expression depicted as mean fluorescence intensity minus isotype control (ΔMFI). (C) CD70-IL15-CAR and CD70-CAR expression was detected by measuring CD27

(legend continued on next page)

without IL-15 (96.4 ± 2.3%, ΔMFI: 383,547.3 ± 308,885.4) (Figure 3C). To then further evaluate the effect of the IL-15 on cytotoxicity, another set of xCELLigence experiments were performed. Additionally, NK-92 cells electroporated with an equimolar concentration of IL-15 mRNA alone, hereafter referred to as IL-15 mRNA, were included. Neither the IL-15 mRNA (1.6 ± 1.2%) nor the MSLN-CAR NK cells (8.2 ± 3.1%) alone were able to achieve improved cytolysis compared to control NK cells (8.2 ± 2.9%) (Figure 3D) in the NK cell-resistant AsPC-1 cell line. However, MSLN-IL-15-CAR NK cells killed significantly better (44.2 ± 14.7%) than the MSLN-CAR NK cells or IL-15 mRNA electroporated NK cells. Since AsPC-1 cells do not express CD70, no significant differences were observed between the CD70-CAR conditions (CD70-IL-15-CAR NK cells: 3.4 ± 3.4%; CD70-CAR NK cells: 2.3 ± 1.7%) (Figure 3D, left).

In line with moderate MSLN expression on PANC-1, both, MSLN-CAR NK cells (23.6 ± 3.8%) and control NK cells (26.4 ± 4.3%) demonstrated similar killing, while the MSLN-IL-15-CAR NK cells killed significantly better (60 ± 2.7%) compared to the MSLN-CAR without IL-15 (Figure 3D, right). However, this was not significantly different from IL-15 mRNA electroporated NK cells (52.6 ± 5.9%), indicating that the observed cytotoxicity was driven by IL-15. Aligned with the high CD70 expression on PANC-1, both CD70-CAR NK cell constructs achieved the highest cytolysis. Both CD70-CAR (65.9 ± 5.4%) and CD70-IL-15-CAR NK cells (70.9 ± 5.9%) showed significantly better cytotoxicity compared to NK cells electroporated with IL-15 mRNA alone (52.5 ± 5.9%). However, no significant difference between the two CD70-CAR NK cell conditions was observed, with only a modest trend toward increased killing when IL-15 was included (Figure 3D, right).

Among the four tested cell lines, RLT-PSC showed the smallest difference in cytotoxicity between conditions with and without IL-15 (Figure 3E, left). NK cells boosted with IL-15 mRNA (48.9 ± 7.5%) killed significantly better than control NK cells (10.8 ± 3.8%). However, there was no significant difference between those IL-15 boosted NK cells and MSLN-CAR-IL15 (59.2 ± 11.3%) or CD70-IL-15-CAR NK cells (59.3 ± 9.4%). Similarly to PANC-1, the MSLN-IL-15-CAR also performed significantly better than the MSLN construct without IL-15 (15.5 ± 3.1%). No difference was observed between both CD70-CAR NK cells conditions. However, both IL-15 containing CAR constructs demonstrated a trend toward higher cytolysis (Figure 3E, left). As hPSC21 showed the highest CD70 expression, both CD70-IL-15-CAR NK cells (87.5 ± 4.3%) and CD70-CAR NK cells (72.9 ± 0.8%) exhibited significantly better cytolysis compared to the MSLN-CAR constructs (MSLN-IL-15-CAR NK cells: 52.5 ± 11.6%; MSLN-CAR NK cells: 22.1 ± 2.7%). Interestingly, IL-15 mRNA electroporated NK cells (71.1 ± 5.5%) showed higher cytolysis compared to the MSLN-CAR-carrying NK cells. Like RLT-PSC and PANC-1 cells, IL-15 mRNA carrying NK cells demonstrated significantly higher killing compared to the control NK cells (17.2 ± 4%). Additionally, MSLN-IL-15-CAR NK cells (52.5 ± 11.6%) killed better than MSLN-CAR NK cells (22.1 ± 2.7%). However, no difference was observed between the CD70-CAR NK cells and the IL-15 mRNA alone. This significant improvement was only evident when both the CAR as well as the IL-15 were present (Figure 3E, right). These data highlight the importance of having both, the CAR and IL-15 to enhance efficacy, especially against NK cell-resistant tumor cell lines. IL-15 improved cytolysis on cell lines with low or moderate MSLN expression (PANC-1, RLT-PSC, and hPSC21) compared to the CAR alone in a CAR-independent mechanism. On CD70^{high} cell lines (PANC-1 and hPSC21), inclusion of IL-15 provided a modest improvement as IL-15-CAR NK cells exhibited a significantly enhanced cytolysis compared to CAR NKs without IL-15. Given the reduced susceptibility of AsPC-1 cells to NK cell-mediated cytotoxicity, we hypothesized that this tumor-intrinsic resistance is driven by an altered expression of NK cell regulatory ligands. Accordingly, we performed a flow cytometry analysis of different markers that are involved in NK cell activation (MICA/B, CD48, PVR via DNAM-1 signaling) or inhibition (HLA-ABC, HLA-E, PD-L1, PVR via TIGIT signaling) (Figures 3F and S2A–S2C). Inhibitory ligands and receptors like MHC-I, HLA-E, and PD-L1 demonstrated variable expression among the four cell lines, with hPSC21 exhibiting the highest levels of HLA-ABC and PD-L1, both in percentage positive cells and ΔMFI (HLA-ABC: 92.8 ± 10.4%, 147 ± 90; PD-L1: 51.7 ± 42%, 5.5 ± 5.4). AsPC-1 cells were overall HLA-ABC$^+$ (89.6 ± 9.6%), but low in ΔMFI (37.1 ± 19) (Figures 3F, 3G, and S2A–S2C) and demonstrated a neutral expression profile, therefore not explaining the resistance seen in the cytotoxicity assays. Activation ligands and receptors like PVR and MICA/B, were most prominently expressed on RLT-PSC with the highest activation/inhibition score (PVR: 83.9 ± 32.2%; 823.2 ± 185.6; MICA/B: 91.1 ± 8.6%; 182.3 ± 43.4) (Figure S3D). Summarized cytolysis indicated that low cytolysis does not necessarily align with high inhibitory

expression on the surface of NK-92 cells using flow cytometry 24 h after electroporation (n = 3). The graph on the left depicts the amount of CAR+ cells while the right graph shows the intensity of CAR expression depicted as mean fluorescence intensity minus isotype control (ΔMFI). (D) Cytolysis data from pancreatic cancer cell lines acquired with the xCELLigence RTCA machine comparing NK cells electroporate with four different CAR constructs (MSLN-CAR, MSLN-IL-15-CAR, CD70-CAR, and CD70-IL-15-CAR), IL15mRNA, or no CAR (NK cells). n = 3–4, error bars represent SEM. (E) Cytolysis data from pancreatic CAF cell lines acquired with the xCELLigence RTCA machine comparing NK cells electroporated with four different CAR constructs, IL15mRNA, or no CAR (NK cells). (n = 3–4) Error bars represent SEM. (F) Bubble plot showing the expression of inhibitory (HLA-ABC, HLA-E, PDL-1, and PVR) or activating NK cell markers (PVR, MICA/B, and CD48) and CAR targets (CD70 and MSLN) across four different PDAC cell lines (AsPC-1, PANC-1, RLT-PSC, and hPSC21). Dot colors represent relative Δ MFI (Z score per ligand, Z score = (x-meanMarker)/SDMarker), and dot size indicates the percentage of positive cells for that marker on the respective cell line. (G) Heatmap summarizing CAR NK cell cytotoxicity (top) and baseline ligand expression (bottom) across four different PDAC cell lines. Cytolysis (%) is shown for five (CAR) NK cells conditions (blue = low cytotoxicity, red = high cytotoxicity). Marker expression is Z-scored ΔMFI (green = low expression, purple = high expression) for inhibitory (HLA-ABC, HLA-E, and PD-L1) and activating (PVR, MICA/B, and CD48) markers. Expression and cytotoxicity were measured in independent experiments; heatmap is descriptive. (n = 3–4) Linear mixed models with Tukey's correction for multiple comparisons were applied to compare the means of cytolysis. $^*p$ <0.05, $^{**}p$ <0.01, $^{***}p$ <0.001, and $^{****}p$ <0.0001.

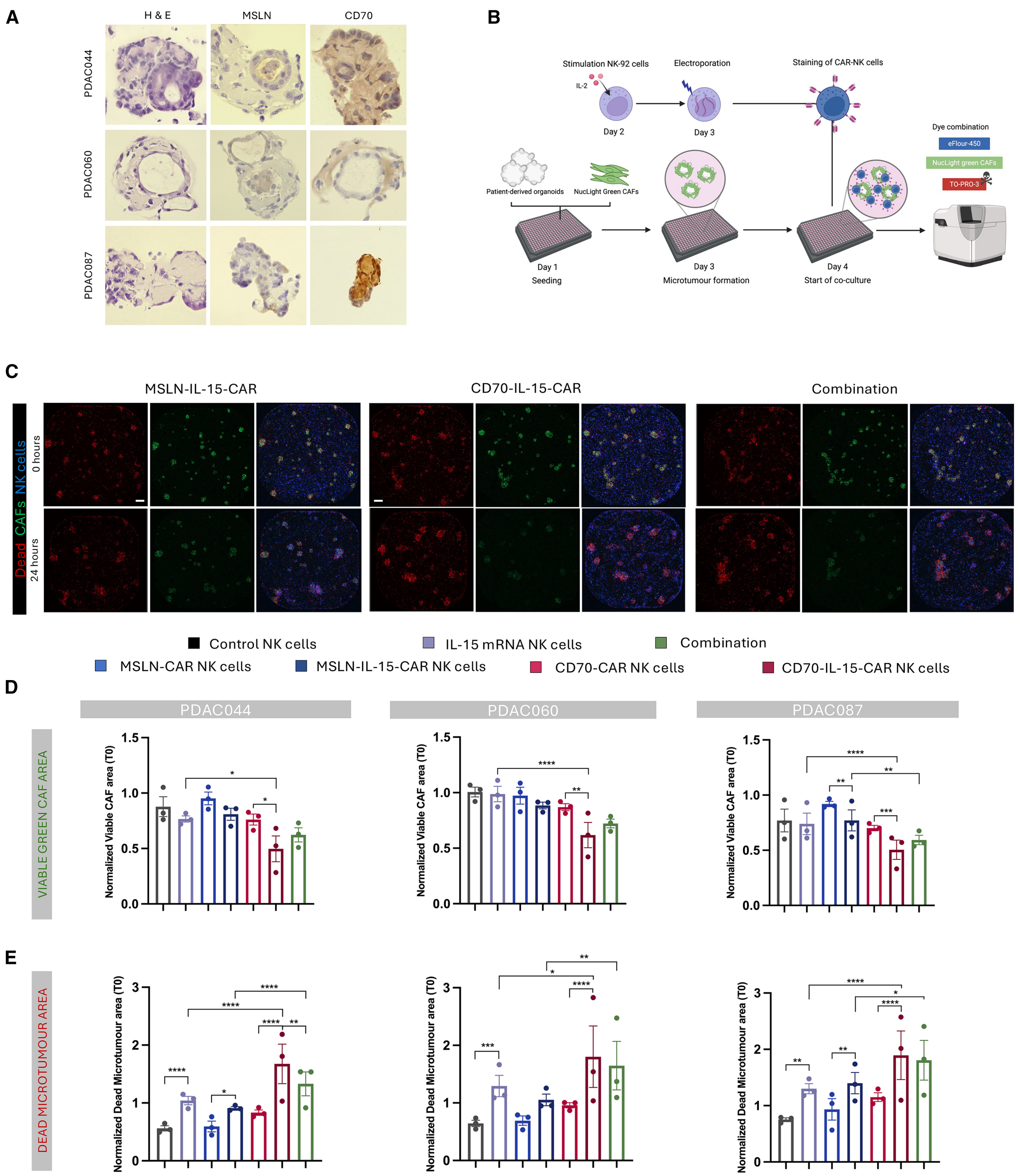

Figure 4. Heterogeneous effects of MSLN- and CD70-CAR NK cells in pancreatic cancer microtumors

(A) Representative images of H&E, MSLN, and CD70 staining of PDAC microtumors, e.g., PDAC patient-derived organoids in co-culture with hPSC21. Magnification 10X. (B) Schematic overview of the experimental setup. (C) Representative images of PDAC044 microtumors at time point 0 and 24 h later after treatment with MSLN-IL-15-CAR, CD70-IL-15-CAR NK cells, or the combination. Scale bars, 200 μm. (D) Quantification of three different PDAC microtumors (PDAC044, PDAC060, and PDAC087). Barplots

(legend continued on next page)

marker expression tested here, nor with a low abundance of activating markers (Figure 3G).

Heterogeneous and patient-dependent effects of MSLN- and CD70-CAR NK cells

To validate the importance of IL-15 and target selection in a clinically more relevant setting, we used patient-derived organoids (PDOs) together with a pancreatic cancer CAF cell line (hPSC21) to create "microtumors". Using IHC staining, we first confirmed the presence of our targets on the microtumors (Figure 4A). Here, PDAC044 microtumors revealed low MSLN and high CD70 expression, PDAC060 microtumors demonstrated low MSLN expression with intermediate CD70 expression. Lastly, PDAC087 microtumors showed low MSLN and high CD70 (Figure 4A). For microtumor co-culture experiments, PDOs and Nuclight green hPSC21 were seeded and 3 days later eFlour-450-stained control NK cells, IL-15 NK cells, MSLN-CAR with and without IL-15 or CD70-CAR NK cells with or without IL-15 were added and imaged using a Zeiss Cell-discoverer 7 microscope (Figure 4B). Twenty-four hours after start of the co-culture, we analyzed viable green CAF area normalized to time point 0 (T0) as an indicator of CAF death. For patient PDAC044 and PDAC060, MSLN-IL-15-CAR NK cells (PDAC044: 0.81 ± 0.01; PDAC060: 0.88 ± 0.05) induced a non-significant reduction of the viable green CAF area compared to MSLN-CAR NK cells (PDAC044: 0.95 ± 0.1; PDAC060: 0.97 ± 0.13). A significant reduction, however, was observed between the CD70-IL-15-CAR NK cells (PDAC044: 0.49 ± 0.2; PDAC060: 0.62 ± 0.2) and the CD70-CAR NK cells (PDAC044: 0.76 ± 0.08; PDAC060: 0.87 ± 0.05) for both microtumors. Additionally, the CD70-IL-15-CAR induced a significantly increased reduction in viable green CAF area compared to IL-15 mRNA electroporated NK cells (PDAC044: 0.76 ± 0.05; PDAC060: 0.98 ± 0.12). In PDAC087, MSLN-IL-15-CAR NK cells showed a significant decrease of viable green CAF area (0.77 ± 0.16) compared to MSLN-CAR NK cells (0.92 ± 0.04). Similarly to PDAC044 and PDAC060, CD70-IL-15-CAR NK cells (0.5 ± 0.15) resulted in a significant reduction of green CAF area in contrast to CD70-CAR NK cells (0.7 ± 0.04). Furthermore, the combination condition (0.59 ± 0.07) differed significantly from MSLN-IL-15-CAR NK cells, but not CD70-IL-15-CAR NK cells (Figures 4C, 4D, and S3A–S3D.) These results demonstrated specific treatments such as CD70-IL-15-CAR NK cells or the combination led to a reduction of viable green CAF area across all three patients.

We also analyzed the red dead microtumor area, normalized to T0 as an indicator of overall cell death, 24 h after the start of the co-culture. This revealed partially similar results to the green CAF area analysis. In PDAC044, both IL-15-expressing CAR constructs (MSLN-IL-15-CAR: 0.91 ± 0.04; CD70-IL-15-CAR: 1.67 ± 0.59) significantly increased the dead microtumor area compared to their respective counterparts without IL-15 (MSLN-CAR: 0.59 ± 0.16; CD70-CAR: 0.83 ± 0.08). We witnessed these differences in almost all patients, except PDAC60, where only CD70-IL-15-CAR NK cells (1.8 ± 0.93) demonstrated a significant decrease compared to CD70-CAR NK cells (0.96 ± 0.08). Furthermore, all three patients showed a significant difference between MSLN-IL-15-CAR NK cells and the combination, whereas the combination increased the dead microtumor area (PDAC044: 1.33 ± 0.4; PDAC060: 1.65 ± 0.73; PDAC087: 1.8 ± 0.61) but not CD70-IL-15 CAR NK cells. Additionally, IL-15 mRNA electroporated NK cells differed significantly from CD70-IL-15 CAR NK cells in their effect on the dead microtumor area (PDAC044: 1.3 ± 0.16; PDAC060: 1.29 ± 0.32; PDAC087: 1.04 ± 0.13) (Figures 4E and S3A–S3E). Analysis of the viable green CAF and the dead microtumor area within the microtumors showed that strongest effects were observed when both IL-15 and the CAR were present in the NK cells. In contrast to our hypothesis, the pooled combination of MSLN-IL-15- and CD70-IL-15-CAR NK cells did not result in a significant increase in cytotoxicity in any of the three patient-derived microtumors. Across both readouts, CD70-IL-15-CAR NK cells showed a consistent trend toward stronger activity than the combination. Notably, changes in dead microtumor area did not always correspond directly to reductions in viable green CAF area, suggesting that the red signal increase is not solely associated with CAF depletion. The magnitude differed between patients, highlighting inter-patient heterogeneity.

To investigate the combined potential of MSLN-IL-15-CAR and CD70-IL-15-CAR NK cells *in vivo*, NSG mice were subcutaneously injected with $MSLN^+$ AsPC-1 and $CD70^+$ hPSC21 (Figures 5A, S4A, and S4B). IHC staining confirmed the presence of MSLN and CD70 within the tumors (Figure 5B). When the tumors reached 100 mm^3, the mice were treated twice (3 days in between) with 5×10^6 control NK cells, MSLN-IL-15-CAR NK cells, CD70-IL-15-CAR NK cells or a combination of MSLN-IL-15 and CD70-IL-15-CAR NK cells or left untreated. We witnessed no survival benefit for the monotherapies or the combination in comparison to the control or effect on tumor growth kinetics (Figures 5C–5F). These results show that CAR and IL-15 engineering may be insufficient to overcome the barriers found in PDAC, and that dual-targeting through a pooled approach may show some additional effects though this is likely highly patient-dependent. Additionally, these findings were acquired using the NK-92 cell line, which differs from primary NK cells in receptor and ligand expression, persistence and tumor-stromal responsiveness. Therefore, future studies will be needed to determine if these observations can be translated using more clinically relevant NK cell products.

DISCUSSION

PDAC therapy faces multiple challenges such as a suppressive TME, high heterogeneity within and between patients and variable antigen

depicting the viable green CAF area 24 h after treatment normalized to the time point 0 (T0) of each well of each patient, respectively. ($n = 3$) (E) Quantification of three different PDAC microtumors (PDAC044, PDAC060, and PDAC087). Barplots depicting the dead microtumor area within microtumors 24 h after treatment normalized to T0 of each well of each patient respectively. ($n = 3$). Linear mixed models with Tukey's correction for multiple comparisons were applied to compare medians. *$p < 0.05$, **$p < 0.01$, ***$p < 0.001$, and ****$p < 0.0001$.

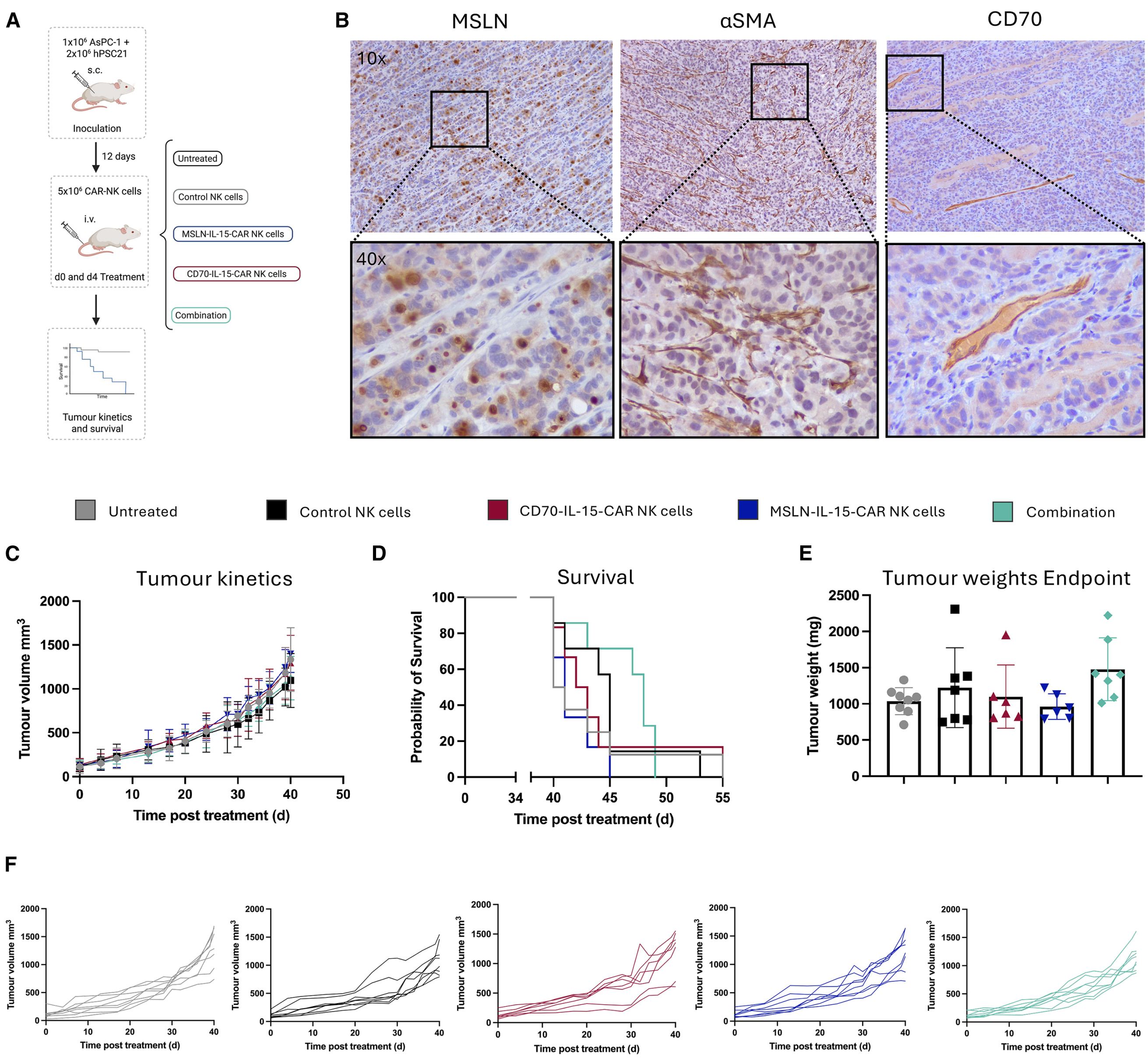

Figure 5. Heterogeneous effects of MSLN- and CD70-CAR NK cells *in vivo*

(A) Schematic overview of the experiment. (B) Representative immunohistochemistry images of FFPE-slides of a AsPC-1 and hPSC21 tumor stained for MSLN, αSMA, and CD70. Images were taken with a 10*X* or 40*X* magnification using the Leica ICC50E microscope. (C) Tumor kinetics over time post-treatment. Error bars represent the mean values ± standard error of the mean. (D) Kaplan-Meier survival curve post-treatment. (E) Tumor weights at endpoint. (F) Separated spider plots of the tumor kinetics over time per treatment group. Mixed model ANOVA was used to compare differences in tumor kinetics and the log rank (Mantel-Cox) test was performed to analyze differences in survival.

density in different tumor regions. Within this context, CAR NK cells present an appealing alternative to autologous CAR T cells, as they are associated with fewer adverse events such as CRS or neurotoxicity.[24] We evaluated whether MSLN and CD70 can serve as therapeutic CAR NK cell targets in pancreatic cancer and whether combined targeting of the stroma as well as tumor cells could lead to a therapeutic benefit. While earlier studies have investigated the role of MSLN-targeting and CD70-targeting CAR T cells as far as of today no study explored the combined therapeutic potential of these two targets in pancreatic cancer, nor has it been explored using CAR NK cell-based approaches.[16,18,25–27]

We analyzed the publicly available TCGA dataset alongside IHC images of PDAC patients, including both primary tumors as well as

liver metastases (not matched). Although our sample size for the liver metastases was relatively small due to limited sample availability, these samples are important and of clinical relevance, as metastatic PDAC samples are hard to collect due to surgical clinical guidelines. Although MSLN-positivity appeared higher and CD70-positivity appeared lower in metastatic samples, these differences were not significant and should be treated with caution due to the low sample size. Nevertheless, this apparent trend of higher MSLN positivity is consistent with previous reports, suggesting a potential association between MSLN expression and metastatic progression in PDAC while another study found no correlation between MSLN expression and lymph node metastasis.[11,13] These differences may be reflected by the heterogeneity and differences in sample size and detection methodology. Supporting its relevance beyond PDAC, high MSLN expression has been associated with invasive potential in other solid tumor types such as gastric cancer or ovarian cancer.[28,29] For CD70, Nakamura et al. reported a higher incidence of metastasis in patients with high CD70 expression rather than low, suggesting a potential association with advanced disease.[20] Similar associations with aggressive tumor behavior have been described in glioblastoma or renal cell carcinoma, although melanoma studies suggest this relationship is tumor-type dependent.[30–32] Together, our findings suggest possible metastatic antigen remodeling in PDAC, with increased MSLN and reduced CD70 expression in metastases. Larger matched primary-metastatic cohorts are required to validate this observation. Nevertheless, the expression of both MSLN and CD70 in pancreatic tumor tissue supports their potential as targets for CAR NK cell therapy. This aligns with other studies that identify both proteins as relevant targets in pancreatic cancer.[13,20,33] However, no association between MSLN expression and reduced survival in PDAC has been observed when using a median cut-off in the TCGA dataset in an exploratory analysis.[34] In contrast, stratification by quartiles revealed a significant difference in overall survival, with patients in the highest 25% quartile of MSLN expression compared to those in the lowest quartile. This finding is in line with the results reported by Cai et al.[35] For CD70, we and others detected no difference in overall survival across the full study cohort.[20] However, co-expression analysis revealed a distinct patient subpopulation characterized by high expression of both MSLN and CD70, which was associated with decreased overall survival. Furthermore, targeting MSLN has been investigated in clinical trials without demonstrating severe adverse events and has been shown to be associated with chemotherapy resistance and promotion of tumor development.[11,16,36,37] Other studies suggest that using MSLN-targeting therapies reduced pancreatic tumors as less MSLN is shed, limiting metastatic outgrowth by preventing MSLN-driven macrophage activation.[38] Moreover, higher CD70 mRNA expression was linked to the upregulation of genes involved in epithelial-to-mesenchymal transition and promotion of tumorigenesis.[20] Notably, there is a difference in protein expression levels and mRNA expression in our results. For example, while MSLN mRNA expression was elevated in a subset of patients, protein-level positivity was detected in a smaller proportion of cases. One could argue that T cell receptor (TCR)-based approaches may be advantageous for targets with higher intracellular or transcript-level expressions, as TCRs can recognize intracellular antigens presented on MHC complexes. Indeed, MSLN has been explored in TCR-signaling approaches, whereas CD70 has been less extensively investigated.[12,39] However, the applicability of TCR-based therapies in PDAC may be limited by the frequent downregulation of MHC-I and therefore impaired antigen presentation.[10] In contrast, CAR NK cells can recognize membrane-associated antigens in an MHC-I independent manner. Importantly, the detection of MSLN on tumor cells and CD70 on CAFs in both primary and metastatic PDAC lesions supports their potential as exploitable targets for the development of targeted immunotherapies.

In line with this, significant anti-tumor activities against MSLN$^+$ and CD70$^+$ PDAC and CAF cell lines were detected using MSLN- and CD70-targeting CAR NK cells *in vitro*, consistent with other papers using similar CAR constructs.[18,40,41] Although recent studies suggest that CAR NK cell function can be further improved by optimizing the CAR architecture by using other costimulatory domains such as CD28, OX40, or 2B4, the ideal design remains context-dependent and may vary according to NK cell source, target antigen, and tumor model.[42–44] In this study, we used a CD28-4-1BB-CD3ζ-based signaling backbone, which has been validated in preclinical and clinical studies.[45–47] Our constructs mediated effective antigen-dependent cytotoxicity, supporting the feasibility of this design. Nevertheless, future studies should directly compare alternative costimulatory, and NK-cell adapted domains, including CD28- or even NKG2D-derived elements.[48,49]

We further demonstrated that antigen density on target cell lines influences CAR NK performance. The MSLN-CAR without IL-15 required high antigen expression to mediate cytolysis, while the CD70-CAR remained active at intermediate levels of CD70 expression. This aligns with published findings showing that antigen abundance is an important factor for CAR NK and CAR T cell function.[50,51] However, the metastatic-derived AsPC-1 cell line exhibited increased resistance toward (CAR-) NK-mediated killing compared to PANC-1, which derived from a primary pancreatic cancer, in line with Oh et al.[52] In addition, the use of the NK-92 cell line may have influenced the observed results. NK-92 cells provide a standardized, easy to culture and highly cytotoxic platform for proof-of-concept CAR NK cell studies, however, they do not fully recapitulate primary NK cell biology. For example, they have a distinct repertoire of activating and inhibitory receptors and lack endogenous CD16 expression, which is needed to mediate antibody-dependent cellular cytotoxicity.[53] Their biggest limitation, due to their origin from an NK cell lymphoma, is the need for irradiation prior to clinical use, potentially limiting their persistence and efficacy.[54] Primary peripheral blood-derived NK cells may better represent mature NK cell biology, including CD16, whereas cord blood-derived NK cells provide an attractive allogeneic source for scalable off-the-shelf CAR NK cell manufacturing due to high inherent proliferative capacity, while NK cells from induced pluripotent stem cells are highly proliferative and can be produced ready to use in biobanks.[53] However, these

primary NK cell platforms introduce donor-dependent variability as well as hurdles with CAR transduction efficacy and follow-up studies should explore our findings in peripheral- or cord-blood-derived NK cells for better clinical relevance.

Furthermore, we explored a potential mechanism that underlies the resistance by profiling activating and inhibitory NK cell marker expression on tumor cell lines, but no consistent pattern was observed. Alternatively, recently published data demonstrated that upregulation of suppressive NK cell markers was only observed after effector cell co-culture, therefore, indicating our used panel should be retested on cells after (CAR) NK cell co-culture.[55] Furthermore, the resistance is likely driven by alternative pathways, including suppressive soluble factors like the TGF-β-axis, which is highly involved in promoting immune evasion in PDAC.[56] In addition, metabolic suppression and, in a more physiological setting, the dense extracellular matrix can restrict effective immune-tumor interactions.[57,58] Furthermore, it would be informative to evaluate the effect of repeated CAR NK cell treatment on tumor-cell outgrowth. Collectively, there are multiple layers of resistance affecting CAR NK cell efficacy, underscoring the complexity of targeting pancreatic cancer. Further investigations into those mechanisms would be valuable; however, it lies beyond the scope of this paper. An additional limitation of the present study is that cytotoxicity was monitored for only 48 h after the start of the co-culture, preventing assessment of whether residual tumor cells regrow at later time points. This is particularly relevant for mRNA-engineered CAR NK cells, where transient CAR expression may require repeated dosing to achieve sustained tumor control.

To overcome resistance, IL-15, a potent pleiotropic cytokine, has been shown to increase persistence and functionality of NK cells.[59] It was noticeable that incorporation of IL-15 into the MSLN-CAR constructs enhanced cytotoxicity against MLSN^{low} cell lines, demonstrating that IL-15 increased the general cytotoxic capacity of NK cells. However, in CD70^{low} AsPC-1 no additional killing was observed with the CD70-IL-15 CAR. Similarly, our microtumor model demonstrated that a combination of CD70-CAR and IL-15 was necessary to achieve a pronounced effect on microtumor killing, visible by an increase in dead microtumor and reduction in viable green CAF area. This highlights that IL-15 alone is insufficient to overcome resistance in such cell lines or more advanced models and that both, CAR expression and IL-15 are required to enhance cytotoxicity. Similarly, MSLN-IL-15-CAR engineered NK cells derived from induced pluripotent stem cells (iPSCs) demonstrated anti-tumor efficacy against multiple solid tumors including PDAC.[60] However, in a glioblastoma model IL-15-armored NK cells induced rapid lethality compared to IL-21-carrying NK cells.[61] This discrepancy underscores that cytokine-driven stimulation is not universally beneficial, and tumor-specific factors are important to consider. Moreover, chronic IL-15 stimulation can itself promote NK cell exhaustion, reducing effector function over time.[62] Importantly, our approach uses mRNA electroporation to transiently deliver the CAR construct with the IL-15 into the NK cells. The short-lived expression minimizes the risk of prolonged IL-15-mediated exhaustion. When PDAC-bearing mice were treated with the different IL-15 bearing CAR constructs, no toxic side effects were detected (Figure S4). Importantly, the choice of cytokine support needs to be tailored to the tumor context and future work to compare the transient versus sustained CAR and cytokine expression should be investigated.

To address additional tumor resistance mechanisms such as tumor escape, antigen relapse and intratumoral heterogeneity, dual-targeting CAR or multitargeting CARs have been developed to simultaneously target two or more tumor-associated antigens to enhance tumor recognition and reduce the likelihood of antigen-negative escape. Considerable dual-targeting strategies have been developed and mostly explored in CAR T cells. Existing engineering approaches include pooled CAR populations, in which individual cells express a single CAR and are subsequently combined; bicistronic constructs enabling the expression of two CARs on one cell; and tandem CARs, in which two antigen-binding domains are incorporated into a single receptor.[63] Dual-targeting strategies have shown promise in PDAC such as tandem HER2/MSLN CAR T cells which displayed enhanced anti-tumor activity in preclinical models or MUC16/MSLN CAR T cells which outperformed monospecific CAR T cells, potentially due to sequential, one antigen-at-a-time binding in heterogeneous tumor settings.[25,64] Our study employed the pooled strategy as a proof-of-concept in which NK-92 cells either expressed an MSLN-IL-15 or CD70-IL-15-CAR and were pooled after electroporation. This approach provided a practical strategy for dual targeting, avoiding additional engineering complexity to determine whether tumor- and stromal-associated antigen targeting improves CAR NK cell function in PDAC. Using a 3D *in vitro* model with PDOs in co-culture with a human CD70^{+} pancreatic cancer CAF cell line (hPSC21), we observed across three different patients a decrease in viable green CAF area, corresponding to CAF cell death, alongside increased dead microtumor area indicative of overall cell death within the microtumor after combination treatment. However, the results did not reach statistical significance compared with other treatment conditions and in some instances were comparable to the CD70-IL-15-CAR alone or even inferior. Work by Ruella et al. demonstrated that dual CD19/CD133 CAR T cells prevented antigen escape in B cell acute lymphoblastic leukemia, with the dual CAR T cells outperforming pooled products, indicating potential limitations of pooled strategies.[65] Potential competition or functional interference between the two CAR NK cell populations should also be considered as a reason for the missing effect. One CAR NK population may survive longer, retain CAR expression more efficiently, or engage more efficiently with tumor cells. Moreover, on dual-positive tumor cells, target accessibility may depend on antigen density, epitope location, and immune synapse formation, which could influence the relative contribution of each CAR NK cell population to cytotoxicity. However, as MSLN and CD70 are not uniformly expressed on the same cells, competition remains to be validated in further work, for example, by using differently labeled CAR NK cell populations and track their tumor engagement.

Consistent with this, transient CAR expression may further impair cytotoxicity. Following elimination of $CD70^+$ CAFs by the CD70-IL-15 CAR NK cells, the remaining MSLN-IL-15-CAR NK cells may exhibit insufficient CAR expression to sustain anti-tumor activity. Notably, cytotoxicity was investigated 24 h after start of the coculture, corresponding to 48 h post-electroporation, where CAR expression was found to decline markedly. Consequently, a sequential treatment strategy or approaches to ensure sustained CAR expression such as viral transduction using retroviruses or adeno-associated virus type are likely to be more effective.[24,66] While sequential administration of two-CAR NK cells represents an interesting alternative to simultaneous dual-antigen targeting, it introduces several translational challenges. A two-step strategy would require decisions about the order of the products, the time interval between doses and whether both CAR NK products should be given at the same doses and whether the second product is given on a fixed schedule or only after incomplete response. Sequential administrations may also require repeated lymphodepletion, additional hospital visits and monitoring of toxicity, even though the risk associated with CAR NK cells are lower compared to CAR T cells. The incorporation of a safety switch could be beneficial.[67] To assess the timing of the sequential strategy integration of multiple biomarkers would be required, including persistent expression of the second target by biopsy or imaging, tumor burden by RECIST 1.1-based CT/MRI or functional imaging as well as pharmacokinetic markers such as CAR NK persistence, immune activation, and cytokine changes. Whether the second CAR NK cell product should be administered after failure of the first construct will likely depend on the mechanism of resistance. In case of persistent expression of the second target and low expression of the first, administration is still rational. However, if failure is associated with poor CAR NK expansion or persistence or severe toxicity after administration of the first product the administration of the second CAR product should be reconsidered. While dual targeting may improve antigen recognition and reduce the risk of antigen-negative tumor escape, it expands the potential window for on-target/off-tumor recognition, particularly for antigens that are expressed in low levels in healthy tissues. Therefore, careful antigen selection and safety validation are necessary. In our study, using the pooled CAR approach with mRNA transfection to evaluate whether coverage can enhance sensitivity in the PDAC microenvironment, we did not witness toxic side effects *in vivo*.

Although our 3D co-culture system recapitulates PDAC characteristics better than conventional 2D cell lines, the dual-targeting approach provided only limited additional benefit over monotherapies, underscoring complexity of the model. We observed inter-experiment and inter-patient heterogeneity, reflecting the well-known variability in PDAC. Notably, monocultured hPSC21 exhibited high CD70 expression, while in co-culture with different PDOs, CD70 levels varied. Additionally, IHC staining of the microtumors revealed low MSLN positivity across all three patients, whereas CD70 expression was intermediate to high, providing an additional explanation for the limited efficacy of MSLN-CAR NK cells. These findings further highlight the importance of sufficient antigen expression. Moreover, NK cell-derived soluble factors potentially modulated CAF behavior, increasing resistance as a study reported upregulation of mesenchymal markers in PDAC cells following co-culture with NK cell-conditioned medium.[68] In the NK cell-resistant AsPC-1 cell line, both CAR expression and IL-15 were required to enhance cytotoxicity, however even under those conditions, tumor cell killing was incomplete. This observation suggests that in an even more complex setting such as our 3D microtumor model, additional immunosuppressive mechanisms limit NK cell efficacy. Previous research by our lab highlights that metabolically engineered CD70-IL-15-CAR NK cells showed improved cytolysis of PDAC microtumors depending on their metabolic profiles.[69] This further highlight that patient heterogeneity needs to be considered and therapies need to be tailored to the environment. In addition to antigen targeting and cytokine support, optimizing cellular fitness can be critical for improving therapeutic efficacy.

Our findings obtained in the 3D *in vitro* model were consistent with the *in vivo* results, in which neither monotherapy nor the combination affected tumor volume. Our *in vivo* model combines a tumor cell line (AsPC-1) with pancreatic CAF cells (hPSC21), thereby incorporating a stromal component that is highly relevant to PDAC biology. Given the major contribution of CAFs to the PDAC TME, this co-injection model provides a more representative setting. Although this model would be useful to study sequential administration of MSLN- and CD70-CAR NK cells, this would introduce new variables including treatment order, time interval between therapies, response-guided versus fixed dosing and assessment of changes in antigen expression after the first treatment. Therefore, we consider sequential administration beyond the scope of the present study, which focused on simultaneous/pooled dual targeting. Similarly, the use of transient mRNA electroporation limited CAR expression and constrained NK cell persistence. Additionally, tumors revealed a dense environment upon harvesting at endpoint, potentially impending NK cell infiltration. These observations highlight again the need for additional NK cell engineering strategies or the use of pre-conditioned cytokine-induced memory NK cells which have demonstrated superior cytotoxic capacity compared to NK-92 cells to enhance persistence and infiltration.[70,71] Furthermore, improved donor/source selection may help to reduce variability in CAR NK cell manufacturing.[24,72] Lastly, the cell dose may have also impacted the results. Due to manufacturing constraints, only 5×10^6 (CAR-) NK cells could be administered i.v. rather than the commonly 1×10^7 cells reported in literature.[18,73] Additionally, when pooled CD19/MSLN-CAR T cells were evaluated in a small cohort of three PDAC patients, they presented an acceptable safety profile but failed to demonstrate clinical efficacy, in line with the outcomes observed on our study.[26]

Collectively, although MSLN-IL-15-CAR NK cells and CD70-IL-15-CAR NK cells were designed to enhance activity through increased target engagement and cytokine support, demonstrated in 2D assays, this activity was difficult to translate to patient-derived samples or *in vivo*. Taken together our results emphasize the need for

combinatorial optimization of effector cell engineering, dosing, delivery, and model systems to reach a therapeutic benefit in pancreatic cancer.

MATERIALS AND METHODS

Data sources, processing, and analysis methods of bioinformatics part

TCGA gene expression data of 33 cancer tissues were obtained from the cBioPortal for Cancer Genomics (https://www.cbioportal.org/).[74–76] The TCGA Pan cancer query was used, and then the mRNA expression data (RSE, batch-normalized from Illumina HiSeq RNA Seq.) were retrieved for all tumor samples. Specifically, gene expression levels of MSLN and CD70 were extracted for each cancer types and respective plots were created using R. To further investigate MSLN and CD70 expression in PDAC, respectively, we used GEPIA2, integrating RNA sequencing data from TCGA tumors and GTEx normal tissues.[23] For PDAC analyses, the Expression DIY/Boxplot module was used to compare the expression of genes of interest. Expression values are displayed on a log2(TPM+1) scale. RNA sequencing and clinical data from TCGA PDAC were analyzed in R, only including samples annotated as primary tumors. Gene expression for MSLN and CD70 were extracted and normalized as log2(CPM+1). For single-gene Kaplan-Meier analyses, patients were stratified into low and high expression groups using the lower and upper quartiles, respectively, excluding the middle 50%. Survival differences were assessed using the log rank test. To assess co-expression, tumor samples were plotted using MSLN and CD70 expression values on a log2(CPM+1) scale. Quartile cutoffs were calculated independently. The quartile thresholds are indicated by dashed lines. The same classification was used to compare the overall survival between the groups and significance was evaluated with the log rank test.

Cell lines and culture conditions

The NK-92 and AsPC-1 cell lines were purchased from the German Collection of Microorganisms and Cell Cultures, the PANC-1 cell line from the American Type Culture Collection. The RLT-PSC and hPSC21 CAF cell lines were kindly provided by Prof. R. Jesenofsky (University of Heidelberg, Mannheim, Germany) and Prof. A. Masamune (Tohoku University Graduate School of Medicine, Sendai, Miyagi Perfecture, Japan).[18] AsPC-1 cells were cultured in Roswell Park Memorial Institute medium (Life Technologies) supplemented with 10% fetal bovine serum (FBS; Life Technologies), 2 mM GlutaMAX (Life Technologies), and 1% Penicillin/Streptomycin (P/S, Life Technologies). PANC-1 cells were cultured in Dulbecco-Modified Eagle Medium (DMEM; Life Technologies) supplemented with 10% FBS (Life Technologies), 2 mM L-Glutamine (Life Technologies), 1% P/S (Life Technologies). hPSC21 and RLT-PSC cells were cultured in DMEM/F12 medium supplemented with 10% FBS (Life Technologies), 2 mM L-Glutamine (Life Technologies), and 1% P/S (Life Technologies). hPSC21 were transduced with NucLight Green (4475, Essen Biosciences). NK-92 cells were cultured in GlutaMAX alpha Minimum Essential Medium (α-MEM; Life Technologies), 12.5% horse serum (Life Technologies), 12,5% FBS (Life Technologies), 2 mM L-Glutamine (Life Technologies), 1% P/S (Life Technologies), and 100 IU/mL of IL-2 (ImmunoTools).

Cell cultures were kept in 5% CO_2 in a humidified incubator at 37°C. Mycoplasma testing was performed regularly with the Mycoalert Mycoplasma detection kit (Lonza) and their identity validated by short tandem repeat (STR) profiling.

Patient and healthy donor samples

Formalin-fixed paraffin embedded (FFPE) tissue blocks of 23 PDAC patients were provided by the Antwerp Biobank (Antwerp, Belgium, ID: BE71030031000) and approved by the Ethics Committee of the Antwerp University Hospital-University of Antwerp (UZA-Antwerp) under study reference EC14/47/480 and EC 13/47/469, respectively. Generation and usage of patient-derived PDAC organoids to create microtumors was approved by patients via written informed consent and by the UZA-UAntwerp Ethics Committee under study reference EC14/47/480.

Immunohistochemistry

MSLN and CD70 expression in the TME of PDAC patients was evaluated on 23 tumor and 5 metastasis resections by manual staining for hematoxylin and eosin (H&E), MSLN and CD70 on consecutive sections from FFPE tissue blocks. Five micrometer-thick sections were baked for 2 h at 60°C and exposed to heat-induced epitope retrieval solution by incubation in Envision FLEX-antigen retrieval solution (DAKO; 20 min). To block endogenous peroxidase activity, 3.5% hydrogen peroxide was added to the slides for 10 min. After blocking with 5% Goat serum (Abcam) for 60 min, immunohistochemical staining of MSLN was performed using a monoclonal anti-mesothelin antibody (EPR4509, 1:200, Abcam) for 45 min. Subsequently, the secondary antibody was added (Goat anti-rabbit IgG vector) for 30 min. Immunohistochemical staining of CD70 was performed similarly, using a monoclonal anti-CD70 antibody (cell signaling, E3Q1A, 1:50). Tumor slides from NSG mice were additionally to MSLN and CD70 stained for αSMA following the same protocol using a monoclonal anti-αSMA antibody (DAKO, 1A4, 1:100).

All sections were counterstained with hematoxylin (Merck; 2 min), dehydrated and mounted with expert mounting medium (Cellpath). Tonsils and mesothelioma tissue were used as a positive control for CD70 and MLSN staining, respectively. The staining protocol was the same for slices of PDAC microtumors and mouse tumor sample slides.

Generation of CAR constructs

The CD70-IL-15-CAR construct was composed of the natural antigen recognition domain of the CD27 receptor, a CD27 transmembrane domain, a 4-1BB co-stimulatory domain, a CD3ζ signaling domain, and an IL-15 cytokine stimulation domain.[18] The Mesothelin CAR incorporates the SS1 scFv from a patent (US7081518B1) and is marked with a FLAG tag (DYKDDDDK). Furthermore, the construct is composed of a CD8α hinge domain, a CD8α-based

transmembrane domain, a 4-1BB costimulatory domain, a CD3ζ signaling domain plus the IL-15 cytokine stimulation domain (Figures 2Aand 3A). The finished CAR sequences were sent to ThermoFisher GeneArt, who created the respective plasmids. The CAR was inserted into a pST1 vector (kindly provided by Prof. Uğur Şahin, University Medical Center Mainz, Mainz, Germany). All plasmids were transformed into *Escherichia coli* (Solo Pack Gold Supercompetent cells, Agilent) and selected on Luria-Bertani Agar containing Kanamycin (Sigma-Aldrich), purified using the NucleoBond Xtra Midi Plus EF kit (Macherey Ngel), and linearized with the SapI/LguI restriction enzyme (MSLN, Life Technologies) and PmeI (CD70, Life Technologies). Transcription into mRNA was performed using the mMessage mMachine T7 transcription kit or MEGAscript transcription kit (Life Technologies) and Cap Analog (m7G(5′)ppp(5′)G) (ThermoFisher Scientific) following manufacturer's instructions and the mRNA concentration was measured on the Nanodrop 200c (ThermoFisher Scientific). The IL-15 mRNA was obtained from the Laboratory of Experimental Haematology and in a pST1 vector.

Electroporation

Electroporation of NK-92 cells was performed utilizing the Lonza 4D Nucleofector X Unit (Lonza) using the program CA-137. Twenty-four hours prior to electroporation NK-92 were stimulated with IL-2 (Immunotools) and fresh medium. On the day of electroporation, the cells were harvested, counted, and afterward washed once in PBS (GIBCO) before the pellet was resuspended in P3 solution (Lonza) containing the CAR mRNA or nothing (for the control). Cells in the P3 solution were transferred into strips (maximum volume 25 μL) or cuvettes for larger transfections (maximum volume of 100 μL). The strip or cuvette was placed inside the machine, and the cells were electroporated. Immediately after electroporation, cells were covered with α-MEM medium and placed in the incubator at 37°C for 10 min. Afterward electroporated NK-92 were recovered in NK cell medium without IL-2 for at least 4 h until further use.

Flow cytometry

Surface expression of MSLN and CD70 on AsPC-1, PANC-1, RLT-PSC, and hPSC21 was assessed using flow cytometry. Cells were detached with TrypLE, counted on the TC20 (Biorad) and stained with the respective antibodies staining for MSLN (primary biotin antibody, clone MB, Biolegend; secondary streptavidin PE antibody, Biolegend) or the respective mouse IgG2α, κ isotype control (Biolegend), CD70-PE (Clone ki-24, BD Biosciences), or respective mouse IgG3-PE isotype control (Clone J606, BD Biosciences). A Live-Dead Fixable Near-IR (Thermo Fisher) was included to separate alive and dead cells. Acquisition was performed on the NovoCyte Quanteon (Agilent Technologies). Surface expression of the MSLN-CAR and the CD70-CAR on NK-92 cells after electroporation was determined using flow cytometry. MSLN-CAR NK cells were stained either with a PE-FLAG-tag (clone L5, Biolegend) or the respective rat IgG2α, λ (PE, Biolegend) isotype control. CD70-CAR NK cells were stained with a CD27-PE mAb (clone O323, Biolegend) or the respective isotype control (clone MOPC-21, Biolegend). Live-Dead Fixable Near-IR dye (Thermo Fisher) was included to separate alive and dead cells. Acquisition was performed on the NovoCyte Quanteon (Agilent Technologies) or BD FACS lyric for CAR expression over time (BD Biosciences). For surface expression of inhibitory and activation markers, AsPC-1, PANC-1, RLT-PSC, and hPSC21 were stained with MICA/B (clone 6D4, Biolegend), PVR (clone SKII.4, BD Biosciences), CD48 (clone TÜ145, BD Biosciences), HLA-ABC (clone W6/32, Biolegend), HLA-E (clone 3D12, Biolegend), PD-L1 (clone MIH1, BD Biosciences), and were measured on the Miltenyi MACS Quant Analyzer 10.

MSLN- and CD70-CAR cytotoxicity experiments

Cytotoxicity against MSLN$^+$ (AsPC-1), CD70$^+$ (PANC-1, RLT-PSC, hPSC21) cell lines was measured using the xCELLigence Real-Time Cell Analysis machine (RTCA, Agilent) which records cell viability and growth by impedance measurements. The killing assay with the xCELLigence RTCA was executed as explained previously.[77] In short, cell lines were harvested and counted. Cell density was optimized to ensure cell growth until the end of the assay. Cancer (AsPC-1, PANC-1) and CAF cells (RLT-PSC, hPSC21) were seeded on gold-coated 16-well or 96-well plates and background impedance was measured before seeding of the target cells. After 24 h incubation to allow attachment of the cells to the bottom of the well, effector MSLN-CAR, MSLN-IL-15-CAR, CD70-CAR, CD70-IL-15-CAR NK cells, IL-15 NK cells, or control NK cells were added in the respective effector: target (E:T) ratio (1:1). The impedance signal was monitored over time every 15 min starting from cell seeding to the end 48 h after starting the co-culture. Measurement of the impedance was calculated as % cytolysis using the immunotherapy module. Untreated controls were taken along.

MSLN$^+$ AsPC1-CD70$^+$ hPSC21 xenograft mouse model

In vivo efficacy of MSLN-IL-15-CAR, CD70-IL-15-CAR NK cells, and control NK cells was evaluated in a subcutaneous PDAC xenograft mouse model. Female NSG mice of 6 weeks old were obtained from Janvier Labs and maintained at the Animal Core Facilities of the UAntwerp under specific pathogen-free conditions in individually ventilated cages. All animal procedures were conducted in accordance with, and approval of, the Animal Ethics Committee of the UAntwerp under registration number 2023-19. Mice were subcutaneously injected with 1.0×10^6 AsPC-1 cells and 2.0×10^6 hPSC21 cells resuspended in 100 μL of sterile PBS (Life Technologies). Tumor growth was measured using a digital caliper and tumor area was assessed by using the formula "tumor area = 0.5*tumor length*(2*tumor width)." When tumors reached 70 mm^3, mice were randomized in different treatment groups: (1) untreated, (2) control NK cells, (3) MSLN-IL-15-CAR NK cells, (4) CD70-IL-15-CAR NK cells, and (5) combination of the MSLN-IL-15-CAR and CD70-IL-15-CAR NK cells. The mice were treated with the respective treatment intravenously with two shots of 5.0×10^6 (CAR) NK cells, administered 3 days apart. For the combination treatment, 2.5×10^6 MSLN-IL-15-CAR and 2.5×10^6 CD70-IL-15-CAR NK cells were administered. Mice were sacrificed when tumors reached 1,500 mm^3 and harvested for IHC.

CAR-mediated cytotoxicity using organoids/microtumors

Organoids

PDOs, originating from PDAC tissue of patient samples, were previously established and characterized.[78] Organoids were cultured in Ad-DF+++ (Advanced DMEM/F12; GIBCO), with 1% GlutaMAX (GIBCO), 1% HEPES (GIBCO), 1% penicillin/streptomycin (GIBCO) and supplemented with 0.5 nM WNT Surrogate-Fc-Fusion protein (ImmunoPrecise), 4% Noggin-Fc Fusion Protein conditioned medium (ImmunoPrecise), 4% Rspo3-Fc Fusion Protein conditioned medium (ImmunoPrecise), 1x B27 without vitamin A (Gibco), 10 mM nicotinamide (Sigma-Aldrich), 1.25 mM N-acetylcysteine (Sigma-Aldrich), 100 ng/mL FGF-10 (Peprotech), 500 nM A83-01 (Tocris), and 10 nM gastrin (R&D Systems). For passaging, the organoids were digested to single cells with TrypLE Express (GIBCO) and replated resuspended in >80% ice-cold Cultrex growth factor reduced BME type 2 (R&D Systems) in full organoid medium supplemented with 10 μM Y-27632 (Selleck Chemicals). Small droplets of 20 μL were plated and were incubated inverted for 30 min at 37°C to allow them to solidify.

Microtumors for cytotoxicity assays

Microtumors were established by co-culturing 75 3-day old PDOs, harvested using Organoid Harvesting Solution (Bio-Techne) with 3.000 NucLight green transfected hPSC21 cells, harvested using TrypLE (Life Technologies) in Ad-DF+++ with 1% cultrex in a 384-well ultra-low attachment microplate (Corning) and incubated at 37°C for 3 days to allow microtumors to self-assemble.[18] After 3 days, effector NK cells were harvested and fluorescently labeled with eFlour-450 (1:4000, Thermo Fisher). For this, (CAR)-NK cells were washed once with PBS followed by eFlour-450 staining by incubating them for 10 min at 37°C. After incubation (CAR)- NK cells were washed once with NK cell medium. Subsequently, (CAR)-NK cells were counted and 7,000 effector cells per well were added. For the combination, 3500 MSLN-IL-15-CAR NK cells and 3500 CD70-IL-15-CAR NK cells were added. All wells were stained with 1 μM TO-PRO-3 (T3605, Thermo Fisher) as a cell death marker. After treatment, plates were imaged using a Zeiss CellDiscoverer 7 microscope equipped with an LSM 900 confocal module. Wells were imaged every 6 h under standard culture conditions, 37°C and 5% CO_2, using a 4× dry objective. For each well, eight z stack images were acquired using bright-field and fluorescence channels corresponding to eFluor-450, 405 nm laser, Nuclight Green, 488 nm laser, and TO-PRO-3, 640 nm laser. All experimental conditions were plated in five technical replicates.

Image analysis

Viable CAF area was quantified per well as the total green-positive area within the microtumor after subtraction of the red-positive area overlapping with the green compartment, thereby excluding CAFs co-staining with the cell death marker. This resulted in a total viable CAF area, in μm^2, for each well and time point. To correct for differences in initial CAF seeding density, viable CAF area values were normalized per well to the viable CAF area at T0. This yielded a dimensionless fold-change metric, referred to viable green CAF area. Values below 1 indicate a reduction in viable CAF area relative to baseline, consistent with CAF loss or cell death over time.

Cell death within each microtumor was quantified as the total red-positive area, defined as the summed projected voxel area, in μm^2, of all red fluorescent objects detected within the microtumor boundary at each time point. To account for well-to-well differences in initial microtumor size, the red-positive area was normalized per well to the corresponding value at time point 0 (T0). This generated a dimensionless fold-change metric, referred to as dead microtumor area. Values greater than 1 indicate an increase in cell death relative to baseline.

Data analysis and statistical analysis

For data analysis, Prism version 10 (GraphPad) was used for graphical representation. Additionally, flow cytometry data were analyzed using the Flow Jo v10.8.2 Software (BD Life Sciences). IHC image analysis was performed in Qpath (V0.5.1). For the quantification of DAB+ cells, the total number of cells per slides was determined, and cells were classified as DAB+ when their DAB signal intensity exceeded a threshold value of 0.2 (CD70) or 0.26 (MSLN). The percentage of DAB^+ cells was then calculated by dividing the number of $threshold^+$ cells by the total cell count on the corresponding slide. The analysis of the TCGA data was performed in R Studio V2025.09.2+418.

Statistics were performed using JMP Pro v16.0.0 (SAS Institute Inc.). For statistical analysis of multi-sample comparison linear mixed models with fixed effect post hoc test were used. For all pairwise comparisons, Tukey's multiple comparison correction was applied. These statistical computations were performed in JMP Pro v16.0.0 (SAS institute). To analyze differences in survival between treatment groups, a log rank (Mantel Cox) test was used. Differences were considered significant different if p values <0.05 ($^*p < 0.05$; $^{**}p < 0.01$; $^{***}p < 0.001$; and $^{****}p < 0.0001$). Error bars represent mean values ± standard deviation or standard error of the mean (SD) unless stated otherwise. For downstream analysis, the eight z stack images were converted into a single two-dimensional projection using the weighted average projection method in ZEN 3.8. Image analysis was subsequently performed in Arivis Pro 4.4.0.

DATA AND CODE AVAILABILITY

The datasets generated during and/or analyzed during the current study are available from the corresponding author upon reasonable request.

During the preparation of this work, the author(s) used Microsoft Copilot to improve language and readability of the manuscript. After using this tool/service, the authors reviewed and edited the content as needed and take full responsibility for the content of the publication.

ACKNOWLEDGMENTS

This work was supported by the University of Antwerp Research Fund (BOF, grant number FFB210425). L.G., A.V.D.E., and T.V. have received Emmanuel van der Schueren fellowships from Kom op tegen Kanker (Stand up to Cancer), the Flemish cancer society (grant numbers OZ10547, OZ9845, and OZ10180). J.V.A. receives a postdoctoral fellowship from Stichting Tegen Kanker (Foundation against Cancer; grant number 2025-034). D.C.-D. was supported by a Junior postdoctoral fellowship from the Research Foundation-Flanders (FWO; grant number 12B2W24N). We would also like to thank several

patrons, as part of this research was funded by donations from different donors, including Dedert Schilde vzw, Willy Floren, and the Vereycken family.

AUTHOR CONTRIBUTIONS

All authors contributed to the study conception and design. Material preparation, data collection, and analysis were performed by L.G.; data analysis of the microtumors was performed by C.D.; statistical testing for the microtumor data was performed by J.O.; the first draft of the manuscript was written by L.G.; and all authors commented on previous versions of the manuscript. All authors read and approved the final manuscript.

DECLARATION OF INTERESTS

The authors have no relevant financial or non-financial interests to disclose.

SUPPLEMENTAL INFORMATION

Supplemental information can be found online at https://doi.org/10.1016/j.omton.2026.201316.

REFERENCES

1. Tonini, V., and Zanni, M. (2025). Pancreatic cancer in 2025: Have we found a solution? World J. Gastroenterol. *31*, 111433. https://doi.org/10.3748/wjg.v31.i43.111433.
2. Drougkas, K., Karampinos, K., Karavolias, I., Gomatou, G., Koumprentziotis, I.-A., Ploumaki, I., Triantafyllou, E., and Kotteas, E. (2024). CAR-T Cell Therapy in Pancreatic and Biliary Tract Cancers: An Updated Review of Clinical Trials. J. Gastrointest. Cancer *55*, 990–1003. https://doi.org/10.1007/s12029-024-01054-2.
3. O'Reilly, E.M., Oh, D.-Y., Dhani, N., Renouf, D.J., Lee, M.A., Sun, W., Fisher, G., Hezel, A., Chang, S.-C., Vlahovic, G., et al. (2019). Durvalumab With or Without Tremelimumab for Patients With Metastatic Pancreatic Ductal Adenocarcinoma: A Phase 2 Randomized Clinical Trial. JAMA Oncol. *5*, 1431–1438. https://doi.org/10.1001/jamaoncol.2019.1588.
4. Valkenburg, K.C., de Groot, A.E., and Pienta, K.J. (2018). Targeting the tumour stroma to improve cancer therapy. Nat. Rev. Clin. Oncol. *15*, 366–381. https://doi.org/10.1038/s41571-018-0007-1.
5. Peng, L., Sferruzza, G., Yang, L., Zhou, L., and Chen, S. (2024). CAR-T and CAR-NK as cellular cancer immunotherapy for solid tumors. Cell. Mol. Immunol. *21*, 1089–1108. https://doi.org/10.1038/s41423-024-01207-0.
6. Raje, N., Berdeja, J., Lin, Y., Siegel, D., Jagannath, S., Madduri, D., Liedtke, M., Rosenblatt, J., Maus, M.V., Turka, A., et al. (2019). Anti-BCMA CAR T-Cell Therapy bb2121 in Relapsed or Refractory Multiple Myeloma. N. Engl. J. Med. *380*, 1726–1737. https://doi.org/10.1056/NEJMoa1817226.
7. Jørgensen, L.V., Christensen, E.B., Barnkob, M.B., and Barington, T. (2025). The clinical landscape of CAR NK cells. Exp. Hematol. Oncol. *14*, 46. https://doi.org/10.1186/s40164-025-00633-8.
8. Rossi, F., Fredericks, N., Snowden, A., Allegrezza, M.J., and Moreno-Nieves, U.Y. (2022). Next Generation Natural Killer Cells for Cancer Immunotherapy. Front. Immunol. *13*, 886429. https://doi.org/10.3389/fimmu.2022.886429.
9. Liu, E., Marin, D., Banerjee, P., Macapinlac, H.A., Thompson, P., Basar, R., Nassif Kerbauy, L., Overman, B., Thall, P., Kaplan, M., et al. (2020). Use of CAR-Transduced Natural Killer Cells in CD19-Positive Lymphoid Tumors. N. Engl. J. Med. *382*, 545–553. https://doi.org/10.1056/NEJMoa1910607.
10. Yamamoto, K., Venida, A., Yano, J., Biancur, D.E., Kakiuchi, M., Gupta, S., Sohn, A.S.W., Mukhopadhyay, S., Lin, E.Y., Parker, S.J., et al. (2020). Autophagy promotes immune evasion of pancreatic cancer by degrading MHC-I. Nature *581*, 100–105. https://doi.org/10.1038/s41586-020-2229-5.
11. Hu, J., Wang, J., Guo, X., Fan, Q., Li, X., Li, K., Wang, Z., Liang, S., Amin, B., Zhang, N., et al. (2024). MSLN induced EMT, cancer stem cell traits and chemotherapy resistance of pancreatic cancer cells. Heliyon *10*, e29210. https://doi.org/10.1016/j.heliyon.2024.e29210.
12. Hassan, R., Butler, M., O'Cearbhaill, R.E., Oh, D.Y., Johnson, M., Zikaras, K., Smalley, M., Ross, M., Tanyi, J.L., Ghafoor, A., et al. (2023). Mesothelin-targeting T cell receptor fusion construct cell therapy in refractory solid tumors: phase 1/2 trial interim results. Nat. Med. *29*, 2099–2109. https://doi.org/10.1038/s41591-023-02452-y.
13. Le, K., Wang, J., Zhang, T., Guo, Y., Chang, H., Wang, S., and Zhu, B. (2020). Overexpression of Mesothelin in Pancreatic Ductal Adenocarcinoma (PDAC). Int. J. Med. Sci. *17*, 422–427. https://doi.org/10.7150/ijms.39012.
14. Hassan, R., Bera, T., and Pastan, I. (2004). Mesothelin: A New Target for Immunotherapy. Clin. Cancer Res. *10*, 3937–3942. https://doi.org/10.1158/1078-0432.Ccr-03-0801.
15. Weidemann, S., Gagelmann, P., Gorbokon, N., Lennartz, M., Menz, A., Luebke, A.M., Kluth, M., Hube-Magg, C., Blessin, N.C., Fraune, C., et al. (2021). Mesothelin Expression in Human Tumors: A Tissue Microarray Study on 12,679 Tumors. Biomedicines *9*, 397. https://doi.org/10.3390/biomedicines9040397.
16. Beatty, G.L., O'Hara, M.H., Lacey, S.F., Torigian, D.A., Nazimuddin, F., Chen, F., Kulikovskaya, I.M., Soulen, M.C., McGarvey, M., Nelson, A.M., et al. (2018). Activity of Mesothelin-Specific Chimeric Antigen Receptor T Cells Against Pancreatic Carcinoma Metastases in a Phase 1 Trial. Gastroenterology *155*, 29–32. https://doi.org/10.1053/j.gastro.2018.03.029.
17. Flieswasser, T., Van den Eynde, A., Van Audenaerde, J., De Waele, J., Lardon, F., Riether, C., de Haard, H., Smits, E., Pauwels, P., and Jacobs, J. (2022). The CD70-CD27 axis in oncology: the new kids on the block. J. Exp. Clin. Cancer Res. *41*, 12. https://doi.org/10.1186/s13046-021-02215-y.
18. Van den Eynde, A., Gehrcken, L., Verhezen, T., Lau, H.W., Hermans, C., Lambrechts, H., Flieswasser, T., Quatannens, D., Roex, G., Zwaenepoel, K., et al. (2024). IL-15-secreting CAR natural killer cells directed toward the pan-cancer target CD70 eliminate both cancer cells and cancer-associated fibroblasts. J. Hematol. Oncol. *17*, 8. https://doi.org/10.1186/s13045-024-01525-w.
19. Jacobs, J., Deschoolmeester, V., Zwaenepoel, K., Rolfo, C., Silence, K., Rottey, S., Lardon, F., Smits, E., and Pauwels, P. (2015). CD70: An emerging target in cancer immunotherapy. Pharmacol. Ther. *155*, 1–10. https://doi.org/10.1016/j.pharmthera.2015.07.007.
20. Nakamura, K., Sho, M., Akahori, T., Nishiwada, S., Kunishige, T., Nakagawa, K., Nagai, M., Takagi, T., Terai, T., and Ikeda, N. (2021). Clinical relevance of CD70 expression in resected pancreatic cancer: Prognostic value and therapeutic potential. Pancreatology *21*, 573–580. https://doi.org/10.1016/j.pan.2021.01.013.
21. Alqaisi, H.A., Cohn, D.E., Chern, J.Y., Duska, L.R., Jewell, A., Corr, B.R., Winer, I.S., Girda, E., Crispens, M.A., Dhani, N.C., et al. (2025). Randomized Phase II Study of Bevacizumab with Weekly Anetumab Ravtansine or Weekly Paclitaxel in Platinum-Resistant/Refractory High-Grade Ovarian Cancer (NCI Trial). Clin. Cancer Res. *31*, 993–1001. https://doi.org/10.1158/1078-0432.Ccr-24-3128.
22. Aftimos, P., Rolfo, C., Rottey, S., Offner, F., Bron, D., Maerevoet, M., Soria, J.-C., Moshir, M., Dreier, T., Van Rompaey, L., et al. (2017). Phase I Dose-Escalation Study of the Anti-CD70 Antibody ARGX-110 in Advanced Malignancies. Clin. Cancer Res. *23*, 6411–6420. https://doi.org/10.1158/1078-0432.CCR-17-0613.
23. Tang, Z., Li, C., Kang, B., Gao, G., Li, C., and Zhang, Z. (2017). GEPIA: a web server for cancer and normal gene expression profiling and interactive analyses. Nucleic Acids Res. *45*, W98–W102. https://doi.org/10.1093/nar/gkx247.
24. Marin, D., Li, Y., Basar, R., Rafei, H., Daher, M., Dou, J., Mohanty, V., Dede, M., Nieto, Y., Uprety, N., et al. (2024). Safety, efficacy and determinants of response of allogeneic CD19-specific CAR-NK cells in CD19+ B cell tumors: a phase 1/2 trial. Nat. Med. *30*, 772–784. https://doi.org/10.1038/s41591-023-02785-8.
25. Jiang, F., Zheng, M., Ding, Y., Xiong, F., Liu, X., Zhou, X., Yan, Z., and Luo, J. (2025). Tandem Dual CAR-T Cells Targeting HER2 and Mesothelin Enhance anti-Tumor Effects in Pancreatic Cancer. Cancer Invest. *43*, 594–607. https://doi.org/10.1080/07357907.2025.2548603.
26. Ko, A.H., Jordan, A.C., Tooker, E., Lacey, S.F., Chang, R.B., Li, Y., Venook, A.P., Tempero, M., Damon, L., Fong, L., et al. (2020). Dual Targeting of Mesothelin and CD19 with Chimeric Antigen Receptor-Modified T Cells in Patients with Metastatic Pancreatic Cancer. Mol. Ther. *28*, 2367–2378. https://doi.org/10.1016/j.ymthe.2020.07.017.
27. Hanina, S.A., Park, T., Lopez, M., Rajasekhar, V.K., Mansilla-Soto, J., Haubner, S., Zhao, H., Kogel, F., Nataraj, S., Banerjee, P., et al. (2026). Sensitive CAR T cells redefine targetable CD70 expression in solid tumors. Science *391*, 896–905. https://doi.org/10.1126/science.adv7378.

28. Saha, S., Mukherjee, C., Basak, D., Panja, P., Mondal, P.K., Ghosh, R., Halder, A., Chowdhury, A., Dhali, G.K., Chattopadhyay, B.K., et al. (2023). High expression of mesothelin in plasma and tissue is associated with poor prognosis and promotes invasion and metastasis in gastric cancer. Adv. Cancer Biol. Metastasis *7*, 100098. https://doi.org/10.1016/j.adcanc.2023.100098.

29. Coelho, R., Ricardo, S., Amaral, A.L., Huang, Y.-L., Nunes, M., Neves, J.P., Mendes, N., López, M.N., Bartosch, C., Ferreira, V., et al. (2020). Regulation of invasion and peritoneal dissemination of ovarian cancer by mesothelin manipulation. Oncogenesis *9*, 61. https://doi.org/10.1038/s41389-020-00246-2.

30. Ge, H., Mu, L., Jin, L., Yang, C., Chang, Y.E., Long, Y., DeLeon, G., Deleyrolle, L., Mitchell, D.A., Kubilis, P.S., et al. (2017). Tumor associated CD70 expression is involved in promoting tumor migration and macrophage infiltration in GBM. Int. J. Cancer *141*, 1434–1444. https://doi.org/10.1002/ijc.30830.

31. Inaguma, S., Lasota, J., Czapiewski, P., Langfort, R., Rys, J., Szpor, J., Waloszczyk, P., Okoń, K., Biernat, W., Schrump, D.S., et al. (2020). CD70 expression correlates with a worse prognosis in malignant pleural mesothelioma patients via immune evasion and enhanced invasiveness. J. Pathol. *250*, 205–216. https://doi.org/10.1002/path.5361.

32. Pich, C., Sarrabayrouse, G., Teiti, I., Mariamé, B., Rochaix, P., Lamant, L., Favre, G., Maisongrosse, V., and Tilkin-Mariamé, A.-F. (2016). Melanoma-expressed CD70 is involved in invasion and metastasis. Br. J. Cancer *114*, 63–70. https://doi.org/10.1038/bjc.2015.412.

33. Flieswasser, T., Camara-Clayette, V., Danu, A., Bosq, J., Ribrag, V., Zabrocki, P., Van Rompaey, L., de Haard, H., Zwaenepoel, K., Smits, E., et al. (2019). Screening a Broad Range of Solid and Haematological Tumour Types for CD70 Expression Using a Uniform IHC Methodology as Potential Patient Stratification Method. Cancers (Basel) *11*, 1611. https://doi.org/10.3390/cancers11101611.

34. Hagerty, B.L., Oshi, M., Endo, I., and Takabe, K. (2023). High Mesothelin expression in pancreatic adenocarcinoma is associated with aggressive tumor features but not prognosis. Am. J. Cancer Res. *13*, 4235–4245.

35. Cai, Z., Li, Z., Zhong, W., Lin, F., Dong, X., Ye, H., Guo, Y., Chen, G., Yu, X., Yu, H., et al. (2025). Targeting Mesothelin Enhances Personalized Neoantigen Vaccine Induced Antitumor Immune Response in Orthotopic Pancreatic Cancer Mouse Models. Adv. Sci. *12*, 2407976. https://doi.org/10.1002/advs.202407976.

36. Hassan, R., Cohen, S.J., Phillips, M., Pastan, I., Sharon, E., Kelly, R.J., Schweizer, C., Weil, S., and Laheru, D. (2010). Phase I Clinical Trial of the Chimeric Anti-Mesothelin Monoclonal Antibody MORAb-009 in Patients with Mesothelin-Expressing Cancers. Clin. Cancer Res. *16*, 6132–6138. https://doi.org/10.1158/1078-0432.Ccr-10-2275.

37. Hassan, R., Blumenschein, G.R., Moore, K.N., Santin, A.D., Kindler, H.L., Nemunaitis, J.J., Seward, S.M., Thomas, A., Kim, S.K., Rajagopalan, P., et al. (2020). First-in-Human, Multicenter, Phase I Dose-Escalation and Expansion Study of Anti-Mesothelin Antibody–Drug Conjugate Anetumab Ravtansine in Advanced or Metastatic Solid Tumors. J. Clin. Oncol. *38*, 1824–1835. https://doi.org/10.1200/jco.19.02085.

38. Luckett, T., Abudula, M., Ireland, L., Glenn, M., Bellomo, G., Stafferton, R., Halloran, C., Ghaneh, P., Jones, R., Schmid, M.C., and Mielgo, A. (2024). Mesothelin Secretion by Pancreatic Cancer Cells Co-opts Macrophages and Promotes Metastasis. Cancer Res. *84*, 527–544. https://doi.org/10.1158/0008-5472.Can-23-1542.

39. Ding, J., Webb, L., Patterson, T., Fleury, M., Zieba, A., Horton, H., Hofmeister, R., Gutierrez, D., and Tighe, R. (2021). 203 A membrane-tethered IL-15/IL-15 receptor fusion protein enhances the persistence and efficacy of CD70-targeted TRuC-T cells. Journal for ImmunoTherapy of Cancer *9*, A214. https://doi.org/10.1136/jitc-2021-SITC2021.203.

40. Torchiaro, E., Cortese, M., Petti, C., Basirico', M., Invrea, F., D'Andrea, A., Franco, L., Sangiolo, D., and Medico, E. (2024). Repurposing anti-mesothelin CAR-NK immunotherapy against colorectal cancer. J. Transl. Med. *22*, 1100. https://doi.org/10.1186/s12967-024-05851-y.

41. Yoon, J.H., Yoon, H.-N., Kang, H.J., Yoo, H., Choi, M.J., Chung, J.-Y., Seo, M., Kim, M., Lim, S.O., Kim, Y.J., et al. (2024). Empowering pancreatic tumor homing with augmented anti-tumor potency of CXCR2-tethered CAR-NK cells. Mol. Ther. Oncol. *32*, 200777. https://doi.org/10.1016/j.omton.2024.200777.

42. Acharya, S., Basar, R., Daher, M., Rafei, H., Li, P., Uprety, N., Ensley, E., Shanley, M., Kumar, B., Banerjee, P.P., et al. (2024). CD28 Costimulation Augments CAR Signaling in NK Cells via the LCK/CD3ζ/ZAP70 Signaling Axis. Cancer Discov. *14*, 1879–1900. https://doi.org/10.1158/2159-8290.Cd-24-0096.

43. Wang, Y., Li, J., Wang, Z., Liu, Y., Wang, T., Zhang, M., Xia, C., Zhang, F., Huang, D., Zhang, L., et al. (2024). Comparison of seven CD19 CAR designs in engineering NK cells for enhancing anti-tumour activity. Cell Prolif. *57*, e13683. https://doi.org/10.1111/cpr.13683.

44. dos Santos, M.H., de Azevedo, J.T.C., da Silva Januário, M.E., Schmidt, D.d.F., Tirapelle, M.C., Biggi, A.F.B., Ebrahimabadi, S., Silvestre, R.N., Covas, D.T., Calado, R.T., and Picanço-Castro, V. (2025). 2B4 co-stimulation and dasatinib modulation enhance anti-CD19 CAR-NK-92 cell cytotoxicity. Front. Immunol. *16*, 1675877. https://doi.org/10.3389/fimmu.2025.1675877.

45. Lei, W., Liu, H., Deng, W., Chen, W., Liang, Y., Gao, W., Yuan, X., Guo, S., Li, P., Wang, J., et al. (2025). Safety and feasibility of 4-1BB co-stimulated CD19-specific CAR-NK cell therapy in refractory/relapsed large B cell lymphoma: a phase 1 trial. Nat. Cancer *6*, 786–800. https://doi.org/10.1038/s43018-025-00940-3.

46. Roex, G., Campillo-Davo, D., Flumens, D., Shaw, P.A.G., Krekelbergh, L., De Reu, H., Berneman, Z.N., Lion, E., and Anguille, S. (2022). Two for one: targeting BCMA and CD19 in B-cell malignancies with off-the-shelf dual-CAR NK-92 cells. J. Transl. Med. *20*, 124. https://doi.org/10.1186/s12967-022-03326-6.

47. Freudenberg-Jahn, K., Ben-Horin, I., Murali Shankar, N., Schuldt, M., Ortiz-Montero, P., Loureiro, L.R., Rackwitz, W., Opitz, C., Krutz, L., Breither, T., et al. (2026). Dual CAR-NK cells targeting PD-L1 and ErbB2 (HER2) exhibit cooperative CAR signaling and counteract solid tumor heterogeneity. J. Exp. Clin. Cancer Res. *45*, 127. https://doi.org/10.1186/s13046-026-03722-6.

48. Zhang, P., Feng, X., Niu, X., Liu, Z., Li, M., Liu, M., Yan, D., Zhang, G., and Wan, X. (2025). CD28 is superior to 4-1BB costimulation in generating CAR-NK cells for tumor immunotherapy. Exp. Hematol. Oncol. *14*, 28. https://doi.org/10.1186/s40164-025-00618-7.

49. Li, Y., Hermanson, D.L., Moriarity, B.S., and Kaufman, D.S. (2018). Human iPSC-Derived Natural Killer Cells Engineered with Chimeric Antigen Receptors Enhance Anti-tumor Activity. Cell Stem Cell *23*, 181–192.e5. https://doi.org/10.1016/j.stem.2018.06.002.

50. Stransky, N., Ji, R., Prause, L., Ganser, K., Wels, W.S., Ruth, P., Huber, S.M., and Eckert, F. (2025). Chimeric antigen receptor NK-92 cell function is modulated by HLA class I expression of target cells. iScience *28*, 112523. https://doi.org/10.1016/j.isci.2025.112523.

51. Majzner, R.G., Rietberg, S.P., Sotillo, E., Dong, R., Vachharajani, V.T., Labanieh, L., Myklebust, J.H., Kadapakkam, M., Weber, E.W., Tousley, A.M., et al. (2020). Tuning the Antigen Density Requirement for CAR T-cell Activity. Cancer Discov. *10*, 702–723. https://doi.org/10.1158/2159-8290.Cd-19-0945.

52. Oh, E., Min, B., Li, Y., Lian, C., Hong, J., Park, G.M., Yang, B., Cho, S.Y., Hwang, Y.K., and Yun, C.O. (2019). Cryopreserved Human Natural Killer Cells Exhibit Potent Antitumor Efficacy against Orthotopic Pancreatic Cancer through Efficient Tumor-Homing and Cytolytic Ability (Running Title: Cryopreserved NK Cells Exhibit Antitumor Effect). Cancers (Basel) *11*, 966. https://doi.org/10.3390/cancers11070966.

53. Basar, R., Daher, M., and Rezvani, K. (2020). Next-generation cell therapies: the emerging role of CAR-NK cells. Blood Adv. *4*, 5868–5876. https://doi.org/10.1182/bloodadvances.2020002547.

54. Suck, G., Odendahl, M., Nowakowska, P., Seidl, C., Wels, W.S., Klingemann, H.G., and Tonn, T. (2016). NK-92: an 'off-the-shelf therapeutic' for adoptive natural killer cell-based cancer immunotherapy. Cancer Immunol. Immunother. *65*, 485–492. https://doi.org/10.1007/s00262-015-1761-x.

55. Lee, S.N., Arseneau, R.J., Arnason, T., and Boudreau, J.E. (2026). Effective allogeneic natural killer cell therapy for pancreatic adenocarcinoma avails conserved activating receptors and evades HLA I-driven inhibition. J. Immunother. Cancer *14*, e013796. https://doi.org/10.1136/jitc-2025-013796.

56. Jun, E., Song, A.Y., Choi, J.-W., Lee, H.H., Kim, M.-Y., Ko, D.-H., Kang, H.J., Kim, S.W., Bryceson, Y., Kim, S.C., and Kim, H.S. (2019). Progressive Impairment of NK Cell Cytotoxic Degranulation Is Associated With TGF-β1 Deregulation and Disease Progression in Pancreatic Cancer. Front. Immunol. *10*, 1354–2019. https://doi.org/10.3389/fimmu.2019.01354.

57. Hartupee, C., Nagalo, B.M., Chabu, C.Y., Tesfay, M.Z., Coleman-Barnett, J., West, J.T., and Moaven, O. (2024). Pancreatic cancer tumor microenvironment is a major therapeutic barrier and target. Front. Immunol. *15*, 1287459. https://doi.org/10.3389/fimmu.2024.1287459.

58. Qin, C., Yang, G., Yang, J., Ren, B., Wang, H., Chen, G., Zhao, F., You, L., Wang, W., and Zhao, Y. (2020). Metabolism of pancreatic cancer: paving the way to better anticancer strategies. Mol. Cancer *19*, 50. https://doi.org/10.1186/s12943-020-01169-7.

59. Waldmann, T.A., Dubois, S., Miljkovic, M.D., and Conlon, K.C. (2020). IL-15 in the Combination Immunotherapy of Cancer. Front. Immunol. *11*, 868. https://doi.org/10.3389/fimmu.2020.00868.

60. Jiang, Q., Yu, W., Ma, J., Zhao, M., Zou, J., Mir, S., Zhang, J., Germain, R.N., and Hassan, R. (2025). Robust differentiation of NK cells from MSLN.CAR-IL-15–engineered human iPSCs with enhanced antitumor efficacy against solid tumors. Sci. Adv. *11*, eadt9932. https://doi.org/10.1126/sciadv.adt9932.

61. Shanley, M., Daher, M., Dou, J., Li, S., Basar, R., Rafei, H., Dede, M., Gumin, J., Pantaleón García, J., Nunez Cortes, A.K., et al. (2024). Interleukin-21 engineering enhances NK cell activity against glioblastoma via CEBPD. Cancer Cell *42*, 1450–1466.e11. https://doi.org/10.1016/j.ccell.2024.07.007.

62. Felices, M., Lenvik, A.J., McElmurry, R., Chu, S., Hinderlie, P., Bendzick, L., Geller, M.A., Tolar, J., Blazar, B.R., and Miller, J.S. (2018). Continuous treatment with IL-15 exhausts human NK cells via a metabolic defect. JCI Insight *3*, e96219. https://doi.org/10.1172/jci.insight.96219.

63. Gómez-Melero, S., Hassouneh, F., Vallejo-Bermúdez, I.M., Agüera-Morales, E., Solana, R., and Caballero-Villarraso, J. (2025). Tandem CAR-T cell therapy: recent advances and current challenges. Front. Immunol. *16*, 1546172. https://doi.org/10.3389/fimmu.2025.1546172.

64. Salas-Benito, D., Birocchi, F., Park, S., Ho, C.E., Armstrong, A., Parker, A.L., Bouffard, A.A., Frank, J.A., Kim, E., Kienka, T., et al. (2025). Tandem CAR-T cells targeting mesothelin and MUC16 overcome tumor heterogeneity by targeting one antigen at a time. J. Immunother. Cancer *13*, e012822. https://doi.org/10.1136/jitc-2025-012822.

65. Ruella, M., Barrett, D.M., Kenderian, S.S., Shestova, O., Hofmann, T.J., Perazzelli, J., Klichinsky, M., Aikawa, V., Nazimuddin, F., Kozlowski, M., et al. (2016). Dual CD19 and CD123 targeting prevents antigen-loss relapses after CD19-directed immunotherapies. J. Clin. Invest. *126*, 3814–3826. https://doi.org/10.1172/JCI87366.

66. Naeimi Kararoudi, M., Likhite, S., Elmas, E., Yamamoto, K., Schwartz, M., Sorathia, K., de Souza Fernandes Pereira, M., Sezgin, Y., Devine, R.D., Lyberger, J.M., et al. (2022). Optimization and validation of CAR transduction into human primary NK cells using CRISPR and AAV. Cell Rep. Methods *2*, 100236. https://doi.org/10.1016/j.crmeth.2022.100236.

67. Głowacki, P., and Rieske, P. (2022). Application and Design of Switches Used in CAR. Cells *11*, 1910. https://doi.org/10.3390/cells11121910.

68. Fiore, P.F., Di Pace, A.L., Conti, L.A., Tumino, N., Besi, F., Scaglione, S., Munari, E., Moretta, L., and Vacca, P. (2023). Different effects of NK cells and NK-derived soluble factors on cell lines derived from primary or metastatic pancreatic cancers. Cancer Immunol. Immunother. *72*, 1417–1428. https://doi.org/10.1007/s00262-022-03340-z.

69. Verhezen, T., Van Den Eynde, A., Verstraelen, P., Gehrcken, L., Palmiotto, G., Lau, H.W., De Vos, W.H., Van Der Heijden, S., Brants, L., Melis, J., et al. (2026). DRP1 depletion protects NK cells from hypoxia-induced dysfunction. Redox Rep. *31*, 2626181. https://doi.org/10.1080/13510002.2026.2626181.

70. Terrén, I., Orrantia, A., Astarloa-Pando, G., Amarilla-Irusta, A., Zenarruzabeitia, O., and Borrego, F. (2022). Cytokine-Induced Memory-Like NK Cells: From the Basics to Clinical Applications. Front. Immunol. *13*, 884648. https://doi.org/10.3389/fimmu.2022.884648.

71. Leong, J.W., Chase, J.M., Romee, R., Schneider, S.E., Sullivan, R.P., Cooper, M.A., and Fehniger, T.A. (2014). Preactivation with IL-12, IL-15, and IL-18 induces CD25 and a functional high-affinity IL-2 receptor on human cytokine-induced memory-like natural killer cells. Biol. Blood Marrow Transplant. *20*, 463–473. https://doi.org/10.1016/j.bbmt.2014.01.006.

72. Pfefferle, A., Contet, J., Wong, K., Chen, C., Verhoeyen, E., Slichter, C.K., Schluns, K.S., Cursons, J., Berry, R., Nikolic, I., et al. (2024). Optimisation of a primary human CAR-NK cell manufacturing pipeline. Clin. Transl. Immunol. *13*, e1507. https://doi.org/10.1002/cti2.1507.

73. Liu, E., Tong, Y., Dotti, G., Shaim, H., Savoldo, B., Mukherjee, M., Orange, J., Wan, X., Lu, X., Reynolds, A., et al. (2018). Cord blood NK cells engineered to express IL-15 and a CD19-targeted CAR show long-term persistence and potent antitumor activity. Leukemia *32*, 520–531. https://doi.org/10.1038/leu.2017.226.

74. Cerami, E., Gao, J., Dogrusoz, U., Gross, B.E., Sumer, S.O., Aksoy, B.A., Jacobsen, A., Byrne, C.J., Heuer, M.L., Larsson, E., et al. (2012). The cBio cancer genomics portal: an open platform for exploring multidimensional cancer genomics data. Cancer Discov. *2*, 401–404. https://doi.org/10.1158/2159-8290.Cd-12-0095.

75. Gao, J., Aksoy, B.A., Dogrusoz, U., Dresdner, G., Gross, B., Sumer, S.O., Sun, Y., Jacobsen, A., Sinha, R., Larsson, E., et al. (2013). Integrative analysis of complex cancer genomics and clinical profiles using the cBioPortal. Sci. Signal. *6*, pl1. https://doi.org/10.1126/scisignal.2004088.

76. de Bruijn, I., Kundra, R., Mastrogiacomo, B., Tran, T.N., Sikina, L., Mazor, T., Li, X., Ochoa, A., Zhao, G., Lai, B., et al. (2023). Analysis and Visualization of Longitudinal Genomic and Clinical Data from the AACR Project GENIE Biopharma Collaborative in cBioPortal. Cancer Res. *83*, 3861–3867. https://doi.org/10.1158/0008-5472.Can-23-0816.

77. Baysal, H., De Pauw, I., Zaryouh, H., De Waele, J., Peeters, M., Pauwels, P., Vermorken, J.B., Smits, E., Lardon, F., Jacobs, J., and Wouters, A. (2020). Cetuximab-induced natural killer cell cytotoxicity in head and neck squamous cell carcinoma cell lines: investigation of the role of cetuximab sensitivity and HPV status. Br. J. Cancer *123*, 752–761. https://doi.org/10.1038/s41416-020-0934-3.

78. Le Compte, M., De La Hoz, E.C., Peeters, S., Fortes, F.R., Hermans, C., Domen, A., Smits, E., Lardon, F., Vandamme, T., Lin, A., et al. (2023). Single-organoid analysis reveals clinically relevant treatment-resistant and invasive subclones in pancreatic cancer. NPJ Precis. Oncol. *7*, 128. https://doi.org/10.1038/s41698-023-00480-y.

OMTON, Volume *34*

Supplemental information

IL-15 boosts mesothelin- and CD70-CAR NK cell potency in PDAC without added benefit from dual targeting

Laura Gehrcken, Jasper Ott, Astrid Van den Eynde, Ho Wa Lau, Dana Liu, Christophe Hermans, Tias Verhezen, Stefanie Peeters, Carole Faghel, Philippe Joye, Gils Roex, Diana Campillo-Davo, Eva Lion, Filipe Elvas, Senada Koljenovic, Jorrit De Waele, Vera Hartman, Geert Roeyen, Hans Prenen, Filip Lardon, Christophe Deben, Evelien Smits, and Jonas Van Audenaerde

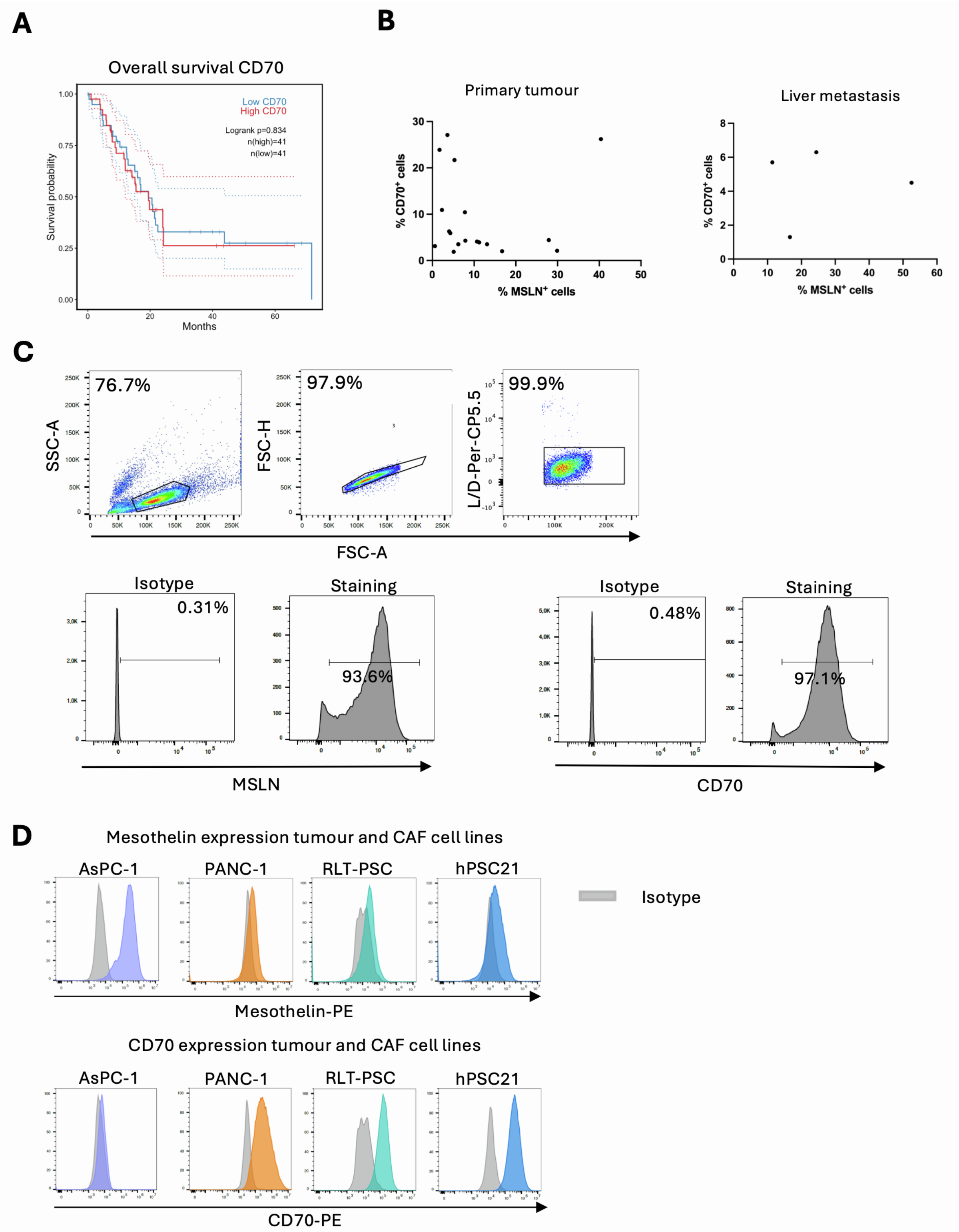

Figure S1 MSLN and CD70 expression in patient samples and PDAC cell lines. *(A) Kaplan-Meier analysis of TCGA-PDAC patients stratified by CD70 expression. Patients in the lower quartile were classified as CD70 low, while patients in the upper quartile were classified as CD70 high. Dotted lines represent 95% confidence intervals. Survival differences were assessed by using the log-rank test. (B) Scatter plot showing the percentage of CD70^{+} cells plotted against the percentage of MSLN^{+} cells across individual tumour (left) and liver metastasis (right) samples, not matched. (C) Representative flow cytometry plots depicting the gating strategy to determine CAR expression 24 h post-electroporation. NK-92 cells were gated for Singlets and then CAR expression analysis on live cells. (Bottom) Representative histograms for MSLN-CAR (left) and CD70-CAR (right) expression in comparison to the corresponding isotype. (D) Representative histograms of MSLN (top) and CD70 (bottom) on the four different cell lines AsPC-1, PANC-1, RLT-PSC and hPSC21 in comparison to the isotype control of the corresponding antibody.*

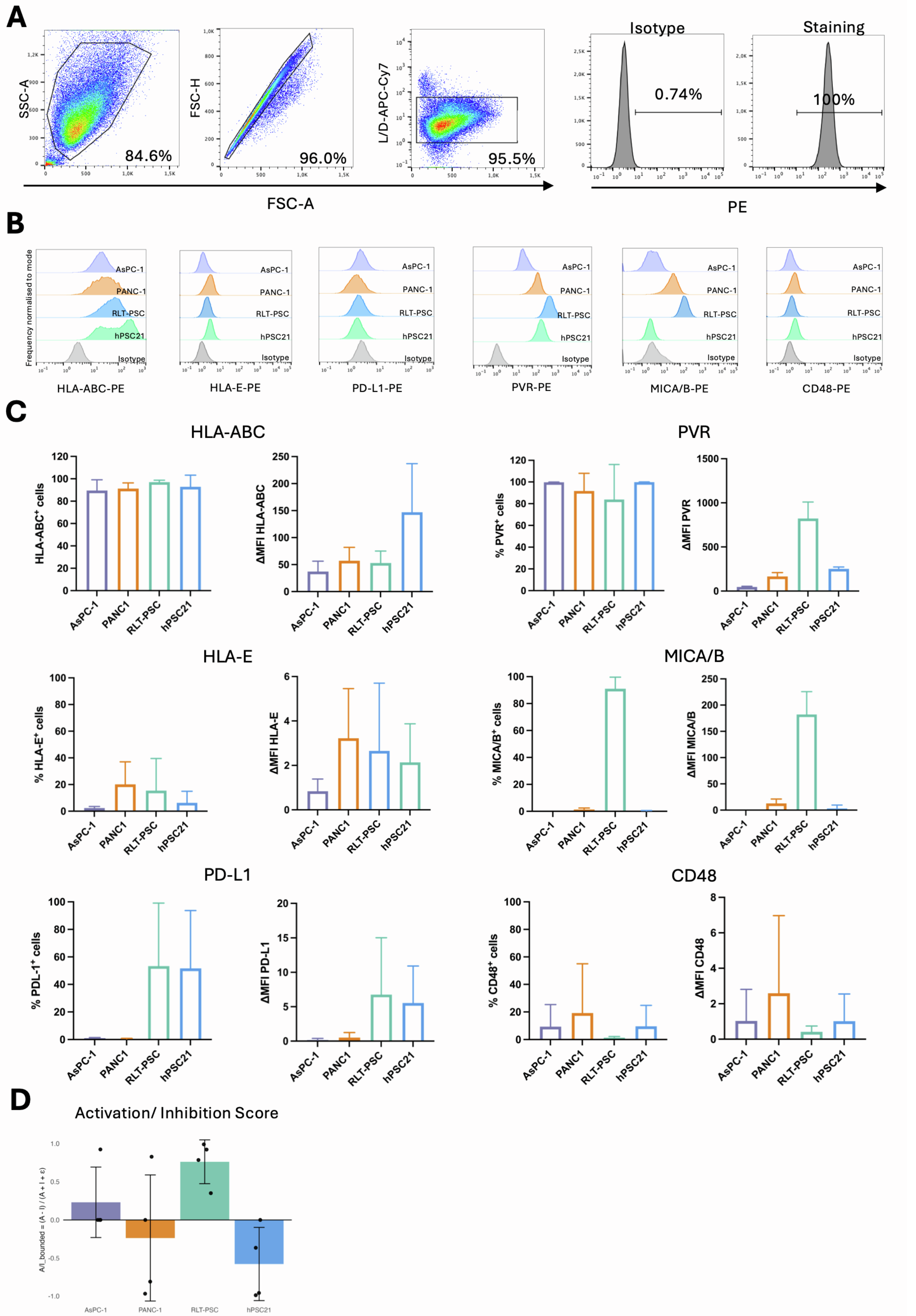

Figure S2 Expression of activating and inhibitory NK cell markers on PDAC tumour and CAF cell lines*. (A) Representative flow cytometry plots depicting the gating strategy to stain different tumour and CAF cell lines for inhibitory and activation marker expression. Tumour and CAF cells were gated for Singlets and then expression of the*

different markers was performed on live cells (left). Representative histograms showing the gating strategy to determine the positivity based on the isotype (right). (B) Representative overlay histograms showing the expression of the different markers (HLA-ABC, HLA-E, PD-L1, PVR, MICA/B, CD48) on the four different cell lines in comparison to the isotype (grey). (C) Individual bar plots for all markers (HLA-ABC; HLA-E; PD-L1, PVR, MICA/B, CD48 investigated on both % positive cells and mean fluorescence intensity minus FMO (fluorescence minus one) control. (D) Bounded antigen index across four cell lines (AsPC-1, PANC-1, RLT-PSC and hPSC21). Bars represent mean AI_bounded values with error bars indicating biological variability. AI_bounded is calculated as (A – I) / (A + I + ε), where A denotes NK-cell activation markers, I denotes inhibitory markers, and ε is a small constant. Positive AI_bounded values indicate a predominance of activation markers, values around 0 reflect a balance between activation and inhibition, and negative values indicate a higher proportion of inhibitory markers.

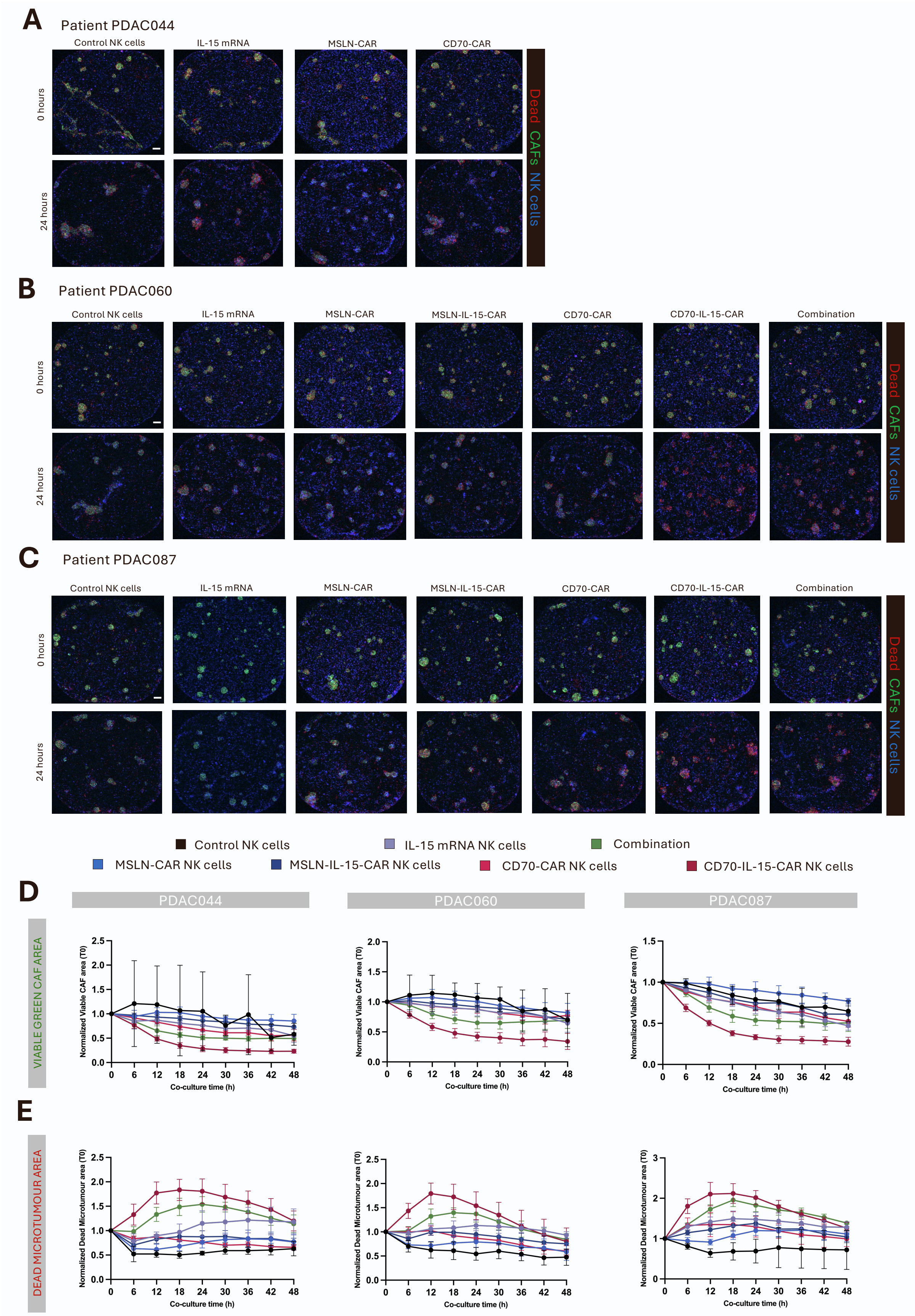

Figure S3 Representative images of PDAC microtumours and quantification of viable green area and dead microtumour area. *(A) Representative images of PDAC microtumours of PDAC044 at timepoint 0 and 24 h later after treatment with control, IL-15 mRNA, MSLN-CAR, CD70-CAR NK cells. Scale bar = 200 µm (B) Representative images of*

PDAC microtumours of PDAC060 at timepoint 0 and 24 h later after treatment with control, IL-15 mRNA, MSLN-CAR, MSLN-IL-15-CAR, CD70-CAR, CD70-IL-15-CAR NK cells or the combination. Scale bar = 200 µm (C) Representative images of PDAC microtumours of PDAC087 at timepoint 0 and 24 h later after treatment with control, IL-15 mRNA, MSLN-CAR, MSLN-IL-15-CAR, CD70-CAR, CD70-IL-15-CAR NK cells or the combination. Scale bar = 200 µm (D) Quantification of viable green CAF area over time in three different PDAC microtumours (PDAC044, PDAC060 and PDAC087). Curves depict area following treatment, normalized to timepoint 0 of each well. n = 3 (E) Quantification of dead microtumour area over time in three different PDAC microtumours (PDAC044, PDAC060 and PDAC087). Curves depict area following treatment, normalized to T0 of each well for each patient. n = 3

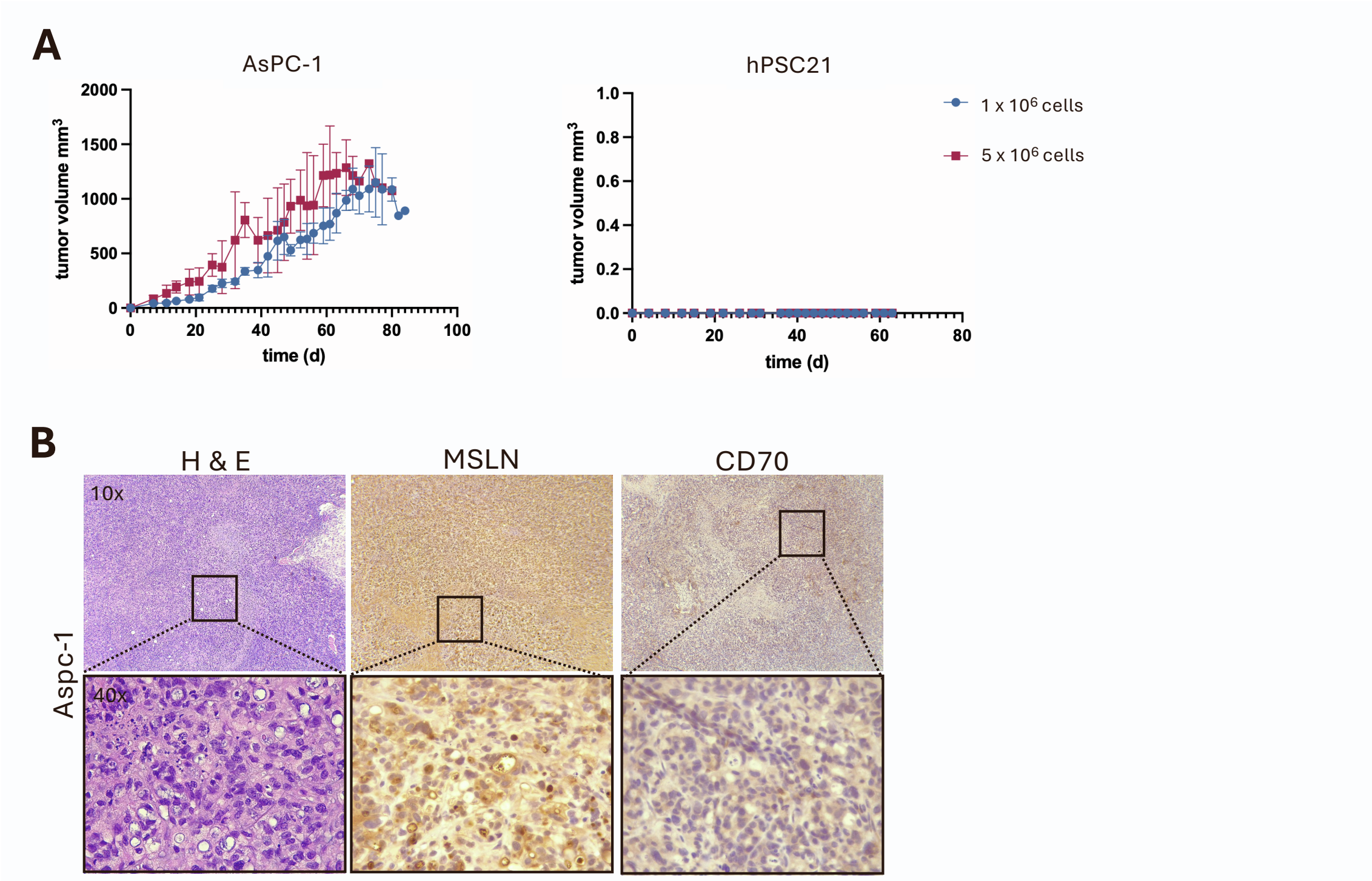

Figure S4 Pilot experiment to establish the mouse model *(A) Tumour growth of NSG mice inoculated with either 1 or 5 x 10^6 AsPC-1 (left) or hPSC21 (right) respectively. (n = 3) (B) Representative IHC images of H&E, MSLN and CD70 staining of 1 x 10^6 AsPC-1 bearing mice. (n = 3)*
